# Facile Synthesis of a Novel Furanic Monomer and Its ADMET Polymerization toward Fully Renewable Functional Polymers

**DOI:** 10.1021/acssuschemeng.4c03498

**Published:** 2024-08-29

**Authors:** Muhammad Kamran, Andrew Kay, Matthew G. Davidson

**Affiliations:** † Institute for Sustainability, University of Bath, Claverton Down, Bath BA2 7AY, U.K.; ‡ Department of Chemistry, University of Bath, Claverton Down, Bath BA2 7AY, U.K.

**Keywords:** biobased, 5-hydroxymethylfurfural (HMF), α,ω-diene monomer, cross-aldol condensation, ADMET, functional polymers, furanic polymers, hydrophobic-coatings

## Abstract

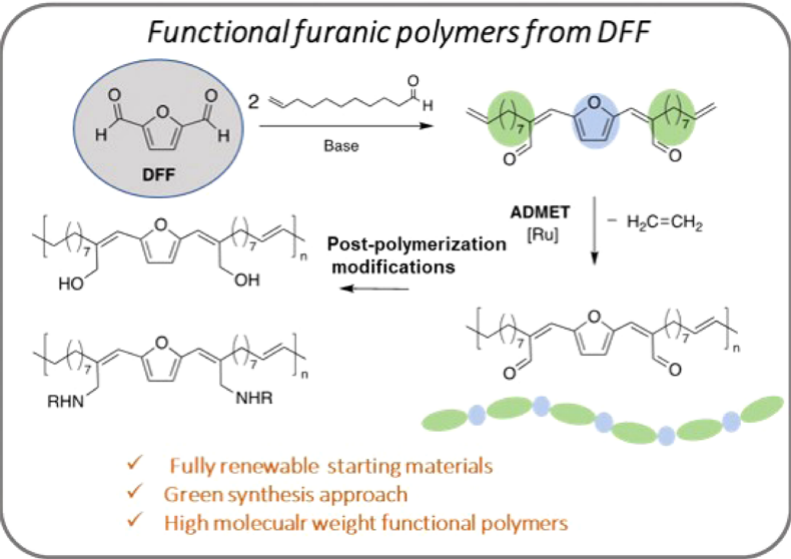

Efficient and sustainable transformation of biomass-derived chemicals to materials with the potential to replace conventional fossil-derived polymers is considered a major challenge. In this work, we disclose the synthesis of a novel furan-based α,ω-diene monomer following a facile, green, and energy-efficient process from fully renewable starting materials. The multifunctional monomer was produced by the base-catalyzed cross-aldol condensation of 10-undecenal (UA) and 2,5-diformylfuran (DFF) under mild conditions, providing the desired product in good yields. By employing the new monomer, fully biobased polymers were prepared in good molecular weights (*M*_n_ up to 31 kg/mol) by acyclic diene metathesis (ADMET) polymerization using Grubb’s second-generation catalysts. The structure–property investigation of the polymers revealed *T*_g_ in the range of −16 to 5 °C, high thermal stability, good hydrophobicity, and photoactive properties. Owning to the presence of amenable functional groups, the resultant polymer was also subjected to postpolymerization modifications. The effect of these modifications on the polymer properties showed enhanced crystallization attributed to hydrogen bonding interactions. This work demonstrates a scalable and environmentally benign approach to access structurally novel and versatile materials exhibiting interesting properties from 100% biobased resources.

## Abstract 2

A novel monomer was prepared from fully renewable starting materials through a facile, energy-efficient, and environmentally green process.

## Introduction

The plastics industry has experienced unprecedented growth in the last few decades. Owing to their many desirable properties, the demand for plastics is forecast to grow substantially and by some estimates may account for around 20% of the global annual oil consumption by 2050.^1,2^ The rapid growth in plastic production and our dependence on petroleum resources have raised environmental concerns. These resources are nonrenewable, supply and prices are subject to fluctuation, and finite reserves are liable to exhaustion.^3^ Biomass on the other hand is entirely renewable and abundant with an estimated production of 1.7 × 10^11^ tons per year.^4^ The renewable biomass-derived feedstocks from lignocellulose, carbohydrates, plant-oils, and terpenes can be utilized for the production of conventional or structurally novel polymers.^5,6^ 5-Hydroxymethylfurfural (HMF), a chemical that can be accessed via the catalytic dehydration of C-6 sugars such as glucose and fructose, has received considerable attention in recent years as a biobased platform molecule. HMF can be transformed into various industrially relevant monomers, chemicals, and biofuels.^7−9^ In particular, 2,5-furandicarboxylic acid (FDCA), produced by the oxidation of HMF, is widely investigated as a renewable alternative to its petroleum-based counterpart terephthalic acid (TPA) in the production of a wide range of materials such as polyethylene furanoate (PEF).^10−15^ In this context, selective oxidation of HMF has been extensively explored for the synthesis of 2,5-diformylfuran (DFF);^16−18^ however, DFF also presents the benefit of being directly accessible from sugars in excellent yields via a one-pot synthesis approach.^19−21^ DFF can also be efficiently synthesized by enzymatic catalysis.^22^ It has found applications in the fields of pharmaceuticals, surfactants, fungicides, furan-urea resins, and vitrimers.^23−26^ However, only a limited number of studies have exploited DFF as a monomer to produce functional thermoplastic polymers.^27,28^

Plant-oils are another crucial renewable source of feedstocks for the chemical industry. They consist of triglycerides of long-chain fatty acids carrying additional functional groups, primarily alkenes, and in some cases, hydroxyl and epoxide groups.^29^ Among plant-oils, castor oil, obtained from the castor bean, has been commercially employed as a feedstock for the production of a variety of monomers used in Nylon-11, Nylon-6,10, and Nylon-12,12.^6,30,31^ 10-Undecenal (UA), employed in this study, can also be prepared by the reduction of 10-undecenoic acid (UNA), obtained from the pyrolysis of ricinoleic acid, the primary constituent of castor oil. By utilizing the alkene bond available in 10-undecenoic acid derivatives, structurally diverse polymers have been synthesized using acyclic diene metathesis (ADMET) polymerization.^32−36^ Moreover, introducing a rigid motif in the ADMET polymers has been shown to render beneficial properties with regard to thermal and mechanical performance of the polymers, thereby providing materials with versatile and tunable properties.^37−41^ Nonetheless, only a few reports have employed α,ω-diene monomers containing furanic moieties for ADMET polymerization. For instance, Wu et al. conducted ADMET polymerization on a furan-based α,ω-diene monomer synthesized by the reaction of FDCA and UNA in the presence of stoichiometric amounts of an activating agent (*N*,*N*’-carbonyldiimidazole).^36^ To address the low reactivity of the acid derivative, Lillie and co-workers employed 10-undecenoyl chloride with 2,5-bis(hydroxymethyl)furan (BHMF) to produce the α,ω-diene monomer, forming HCl as a byproduct.^42^ Therefore, while some promising progress has been made, the development of new and more environmentally friendly, cost-effective, and scalable processes for synthesizing α,ω-diene monomers employing furanic moieties is highly desirable.

Herein, we report the synthesis of a novel multifunctional furanic α,ω-diene monomer using base-catalyzed cross-aldol condensation of DFF and UA conducted under mild conditions. The facile and environmentally benign synthesis approach employing fully renewable starting materials gives the α,ω-diene monomer in good yields with water as the sole byproduct. The monomer was subsequently subjected to ADMET polymerization in the presence of Grubb’s second-generation catalysts, yielding polymers with high molecular weights. The scope of postpolymerization modifications, photoactivity of the resultant polymer, and their impact on the polymer properties were also briefly explored to highlight the potential of this platform chemistry toward sustainable functional materials.

## Results and Discussion

### Synthesis of DFF

2,5-Diformylfuran (DFF, **1**) was successfully synthesized by the partial oxidation of 5-hydroxymethylfurfural (HMF) using manganese(IV) oxide as the oxidant following a modified method described elsewhere (see Figure S1).^43^

### Synthesis of Furan-Based α,ω-Diene Monomers

**Scheme 1:**
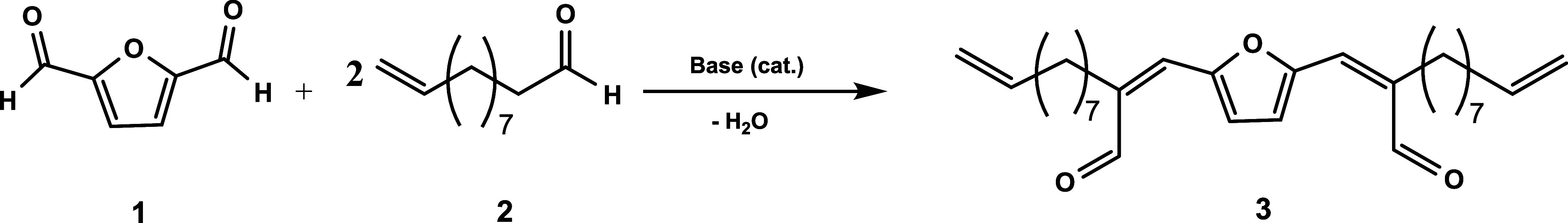
Synthesis of Furan-Based α,ω-Diene Monomer **3** by Cross-Aldol Condensation of 2,5-Diformylfuran **1** and 10-Undecenal **2** under Basic Conditions

Previously, α,ω-diene compounds containing furanic moieties were synthesized either via the incorporation of stoichiometric amounts of an activating agent in a multistep synthesis, or a more reactive acyl-chloride derivative was employed to introduce the terminal alkenes.^36,39^ Both strategies present a significant environmental challenge due to the generation of equivalent quantities of waste byproducts. Herein, we report a facile, inexpensive, and green synthetic approach to produce a novel furanic α,ω-diene monomer **3**. Starting from DFF **1**, **3** was synthesized by the cross-aldol condensation of **1** and 10-undecenal **2** (Scheme 1).^44^ The reaction was initially conducted in the presence of aqueous NaOH as a base catalyst in methanol at room temperature, providing **3** in good yields within 1.5 h (Table 1, entry 1). The isolated yield improved to 78% at multigram scale (Table 1, entry 6; see the Supporting Information for details). Since **2** can readily be obtained from castor oil, this constitutes **3** as a fully renewable monomer. Moreover, the reaction produces water as the sole side product, and reaction/purification solvents can also be recycled after purification via distillation. Thus, qualitatively, the synthesis of monomer **3** represents a green and environmentally benign synthesis. More quantitatively, green metrics relating to the synthesis of monomer **3** have been calculated, together with those for previously reported routes to furan-based α,ω-diene monomers (see Supporting Information Section S3 for details). Metrics for the synthesis of **3** include 92% atom economy, 100% carbon efficiency, process mass index (PMI) of 11.8, simple e-factor of 0.46, and 72% reaction mass efficiency. These metrics represent a significant improvement over previously reported routes to related monomers (Table S2).

**Table 1:** Effect of Various Base Catalysts and Reaction Conditions on Cross-Aldol Condensation Product Yields^a^

entry	solvent	base	temperature/duration	yield^b^ (%)
1	MeOH	NaOH	rt, 1.5 h	71 (68^c^)
2	EtOH	NaOH	rt, 1.5 h	66 (56^c^)
3	MeOH	K_2_CO_3_	rt, 5 h	61
4	H_2_O	NaOH	rt, 5 h	28
5	d	NaOH	rt, 5 h	18
6^e^	MeOH	NaOH	rt, 1.5 h	78^c^
7	MeOH	CaO	40 °C, 5 h	44
8	MeOH	MgO	40 °C, 24 h	2

a Typical reaction conditions: DFF **1** (108 mg, 1.0 equiv), solvent (5 mL), base (1 M, 0.3 mL), UA **2** (0.33 mL, 2.01 equiv); see the Supporting Information for details.

b Calculated from ^1^H NMR analysis using maleic acid as a calibrant.

c Isolated yield obtained after column chromatography.

d Reaction conducted without the solvent.

e Reaction conducted on a multigram scale.

Next, we tested other solvents and base catalysts for the reaction (Table 1). The reactions performed under aqueous and solvent-free conditions resulted in lower yields, possibly due to the limited miscibility of starting material **2**. K_2_CO_3_ was found to be an excellent and mild alternative to NaOH. From the two heterogeneous base catalysts tested, CaO gave better results with a moderate yield of 44% within 5 h at 40 °C. Overall, in the present work, cross-aldol condensation performed using NaOH as a base catalyst in methanol was found to be the most effective route to access **3**.

**Figure 1:**
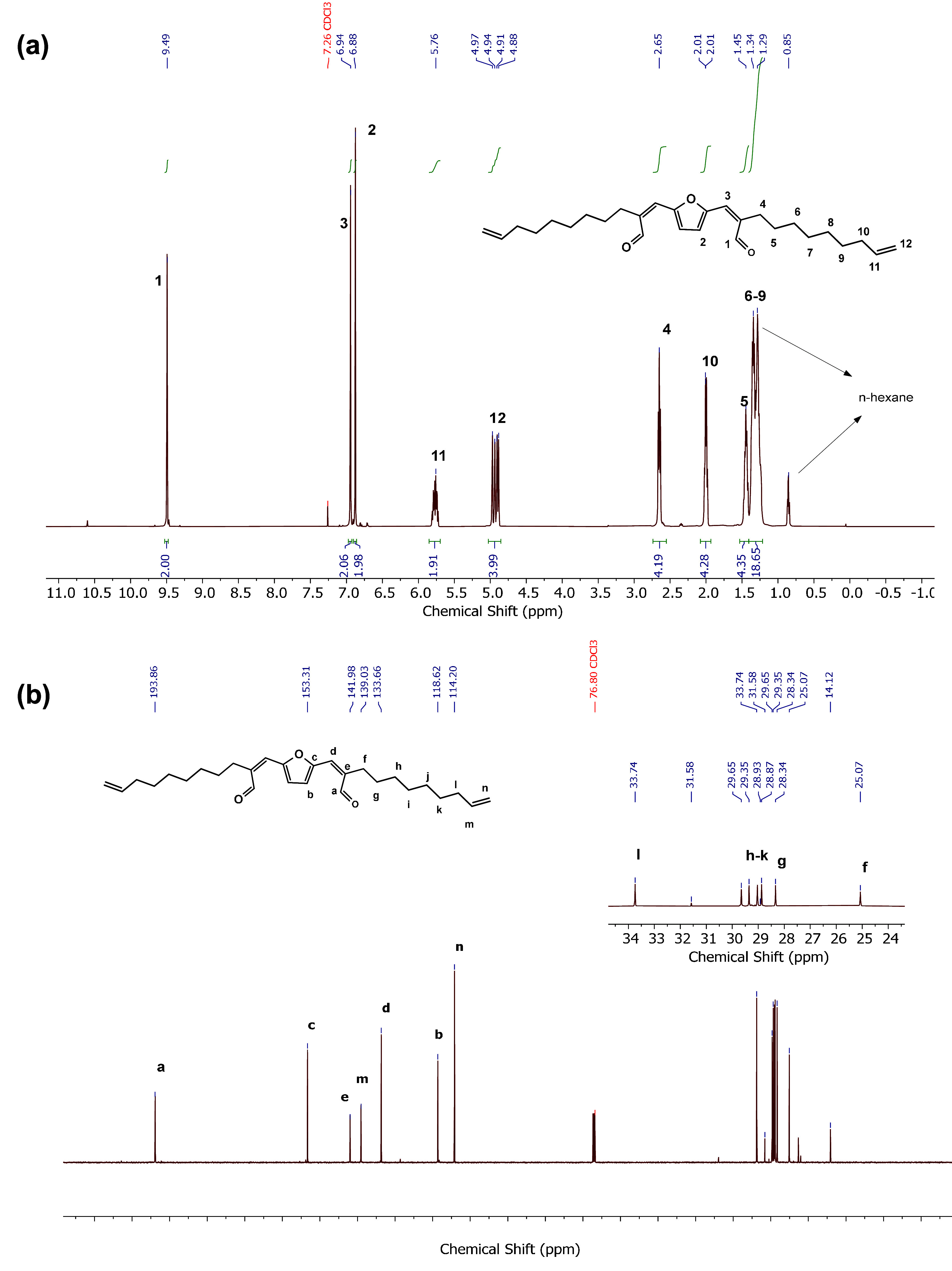
Structural elucidation of monomer **3** by NMR analyses conducted in CDCl_3_ at 25 °C: (a) ^1^H NMR and (b) {^1^H} ^13^C NMR.

The chemical structure of **3** was confirmed by NMR analyses and mass spectrometry (Figures S2–S3 and S6). The ^1^H and ^13^C NMR spectra with the corresponding assignments are depicted in Figure 1. Notably, the reaction conditions employed in the current study significantly limit the undesired self-aldol condensation of **2**, as observed by the ^1^H NMR spectrum of the crude reaction mixture.

**Figure 2:**
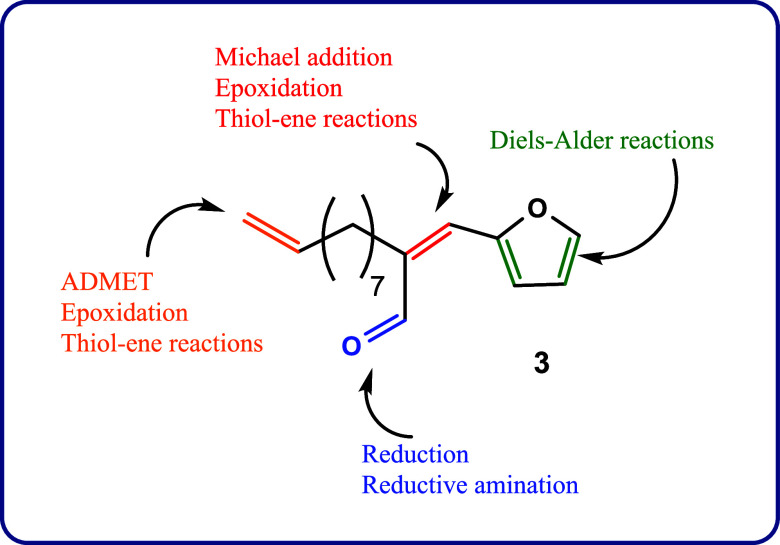
Potential chemical modification pathways accessible for compound **3** (only half of the structure is presented for clarity).

Due to the diversity of functional groups available, compound **3** can be subjected to various facile chemical transformations to design novel materials with targeted properties and end-use applications. As a nonexhaustive example, some of these potential transformations are illustrated in Figure 2. The present work is dedicated to exploring the acyclic diene metathesis (ADMET) polymerization of monomer **3** and subsequent postpolymerization modifications of the resultant polymers.

### ADMET Polymerization

**Figure 3:**
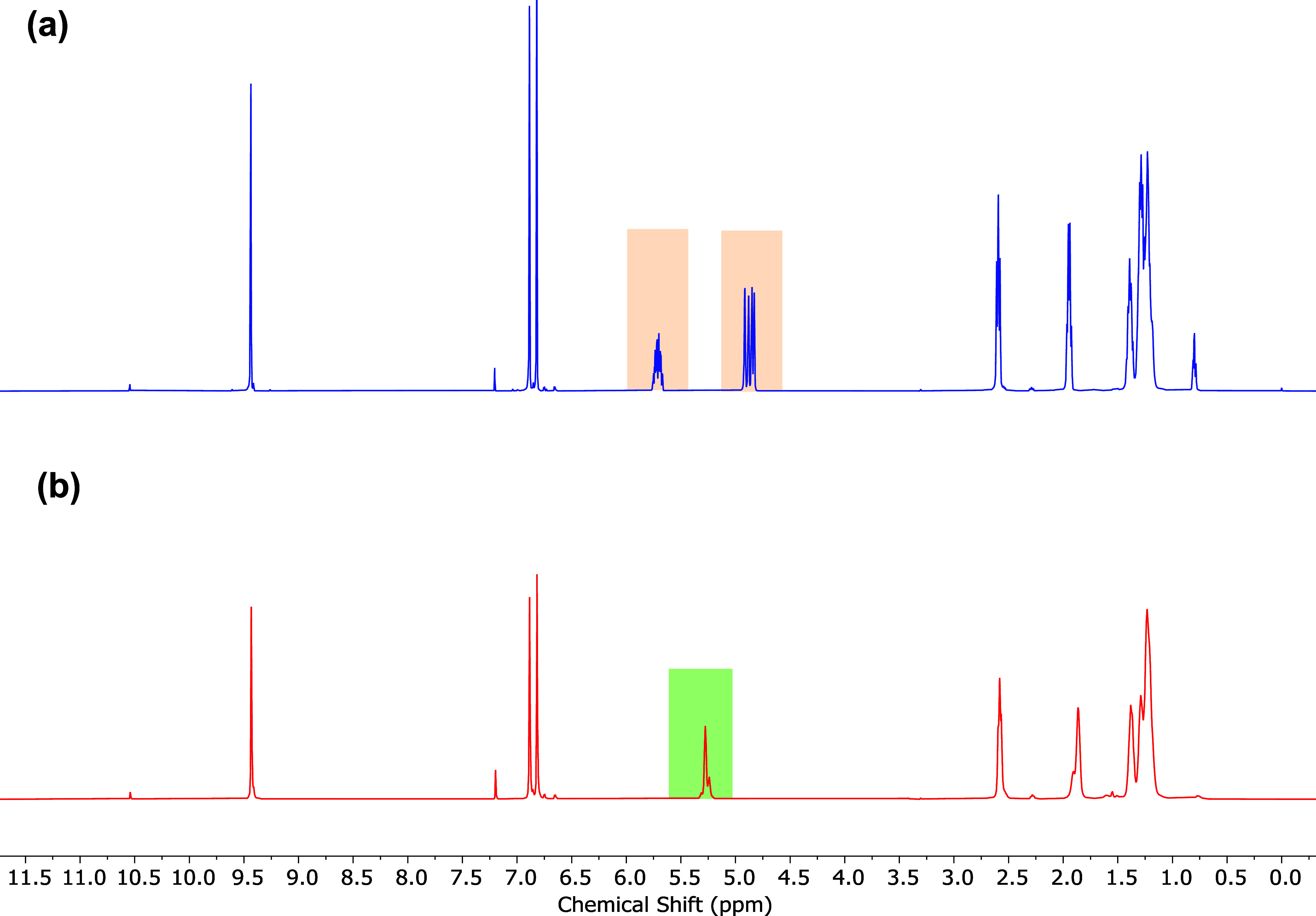
ADMET polymerization of furan-based α,ω-diene monomer **3**: (a) ^1^H NMR of monomer **3** with terminal olefin protons highlighted and (b) ^1^H NMR of the ADMET polymer (PF4, Table 2) exhibiting disappearance of terminal alkene protons and appearance of the newly formed internal alkene protons.

**Scheme 2:**
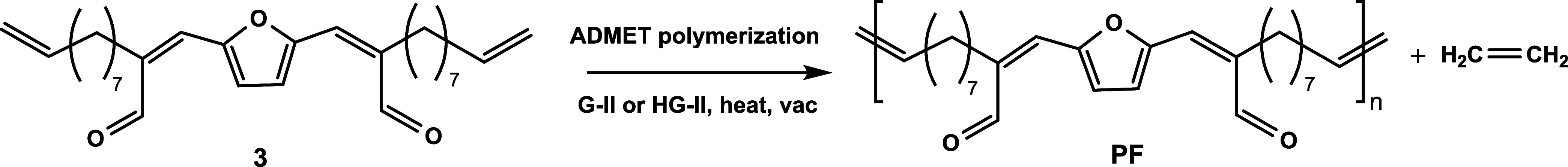
Schematic Representation of ADMET Polymerization of Furan-Based α,ω-Diene Monomer **3**

The ADMET polymerization and reaction optimization of monomer **3** was first carried out in the presence of Grubb’s second-generation (G-II) catalysts, and later, Hoveyda–Grubbs second generation (HG-II) was also tested to afford the corresponding unsaturated polymer (Scheme 2). The reaction was performed in the absence of any solvent using an overhead stirrer. A dynamic vacuum was applied to remove the ethylene formed as the condensation side product. To confirm the chemical structure of the novel AMDET polymer, detailed NMR analyses were conducted. From ^1^H NMR, the formation of the AMDET polymer was substantiated by the disappearance of the terminal olefin protons at 4.94 and 5.76 ppm and the appearance of a new signal at the 5.30–5.34 ppm region assigned to the newly formed alkene protons within the polymer chain (Figure 3; for detailed assignments, see Figure S4). The olefin signal due to the polymer showed a major peak at 5.34 ppm and an adjacent minor signal at 5.30 ppm. As previously reported by other groups, the splitting of this olefin signal suggests some undesired isomerization with predominantly *trans* configuration for both catalytic systems utilized in the present work.^35,45^ The effect of the reaction temperature on the extent of isomerization was also evident with polymers produced at a higher temperature (90 °C) exhibiting a slightly higher degree of double-bond isomerization in the presence of a G-II catalyst (Figure S5).^46^

Monomer conversion was evaluated by ^1^H NMR on crude polymer samples taken before polymer dissolution in tetrahydrofuran (THF). All polymer samples were found to be completely soluble in THF and CDCl_3_, demonstrating excellent tolerance of the catalysts to the presence of conjugated double bonds and aldehyde functionalities. Polymer samples were purified by precipitation into cold methanol after quenching the reaction with ethyl vinyl ether (EVE). Further characterizations, such as GPC, DSC, TGA, and tensile measurements, were all conducted on the purified polymers.

The effect of ADMET polymerization conditions and catalyst loadings was investigated using the G-II catalyst (Table 2, PF1–PF6; see also Figure S10). Polymerization performed at 40 °C and 0.5 mol % G-II catalyst afforded a low molecular weight polymer, possibly due to the incomplete monomer conversion. Increasing the temperature to 55 °C resulted in quantitative monomer conversion in a shorter reaction time, and a polymer having an *M*_n_ of 11 kg/mol was obtained. Reducing the catalyst loading to 0.25 mol % had an adverse effect on the monomer conversion and molecular weight, which could be attributed to the lower reaction rate. However, by increasing the temperature to 80 °C and reaction time to 16 h, a notable increase in the molecular weight was observed at 0.25 mol %. This resulted in a polymer of *M*_n_ 18.7 kg/mol, having a higher dispersity (*Đ* = 3.1). Further increase in temperature to 90 °C improved the molecular weight to 20.7 kg/mol (*Đ* = 2.9) using a 0.5 mol % catalyst.

**Table 2:** Properties of Polymers Synthesized by ADMET Polymerization Using Grubb’s Second-Generation Catalysts (G-II, HG-II)^a^

cat.	polymer	cat.^b^ (mol %)	temperature (°C)	time (h)	*M*_n_^c^ (kg/mol)	*M*_w_^c^ (kg/mol)	*Đ*	conv.^d^ (%)	yield^e^ (%)
G-II	PF1	0.50	40	10	4.2	6.8	1.6	91	72
	PF2	0.50	55	5	10.9	25.2	2.3	100	88
	PF3	0.25	55	5	3.5	5.2	1.5	80	86
	PF4	0.25	80	16	18.7	58.9	3.1	99	94
	PF5	0.50	90	6	12.8	28.1	2.2	100	96
	PF6	0.50	90	16	20.7	60.8	2.9	100	96
HG-II	PF7	1.0	80	16	16.3	33.1	2.0	98	97
	PF8	0.50	80	16	31.2	78.3	2.5	99	77
	PF9	0.25	80	16	18.6	53.8	2.9	99	91
	PF10	0.25	90	16	19.9	58.5	2.9	99	89
	PF11	0.25	90	6	20.6	51.3	2.5	98	93
	PT1^f^	0.50	90	16	21.2	61.5	2.9	98	85

a Typical reaction conditions: Monomer **3** (1.80 g, 1.0 equiv), G-II catalyst (0.25 mol %), 90 °C, 16 h, under dynamic vacuum 1–2 mbar. See the Supporting Information for details.

b Catalyst loading relative to the monomer concentration.

c Determined on purified polymer samples by GPC analysis using THF as the mobile phase and relative to polystyrene calibration standards.

d Measured by ^1^H NMR following the relative disappearance of the terminal olefin signals at 4.94 ppm.

e Determined as an insoluble fraction after precipitation in cold methanol.

f Polymer synthesized from the monomer produced by the cross-aldol condensation of terephthalaldehyde (TAA) and UA; see the Supporting Information for synthetic details.

HG-II catalyst was also found to be active for the current polymerization system (Table 2, PF7–PF11; see also Figure S11). At 0.5 mol % HG-II catalyst loading and for the polymerization performed at 80 °C, a significantly high molecular weight polymer (*M*_n_ ∼ 31 kg/mol) with a dispersity of *Đ* = 2.5 was obtained. Interestingly, due to the enhanced activity of the HG-II catalyst system compared to G-II, at 0.25 mol % catalyst, an *M*_n_ 20 kg/mol polymer was produced within 6 h, therefore considerably reducing the reaction time and energy input for the polymerization process. Polymers with both catalyst systems were produced in good yields (>70%).

**Scheme 3:**
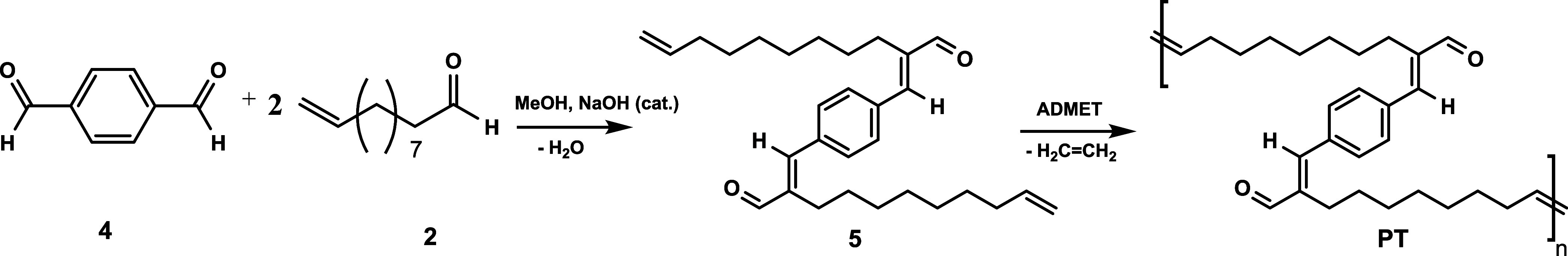
Synthesis Scheme Representing the α,ω-Diene Monomer **5** from Terephthalaldehyde **4** by Cross-Aldol Condensation Reaction and Subsequent AMDET Polymerization

To study the effect of the central aromatic core on the crystallization and thermal properties, the partially biobased counterpart of monomer **3** was prepared by the cross-aldol condensation of terephthalaldehyde (TAA) **4** and 10-undecenal **2** (Scheme 3; see also Figures S6, S7, and S9 for details). Interestingly, the change from furan to benzene ring in the central aromatic core led to an increased crystallinity of the monomer, allowing for simpler purification as monomer **5** was isolated as a white crystalline solid. The monomer **5** was also successfully polymerized using the HG-II catalyst (Table 2, PT1), ensuing a polymer with an *M*_n_ of 21 kg/mol.

### Polymer Characterization

**Figure 4:**
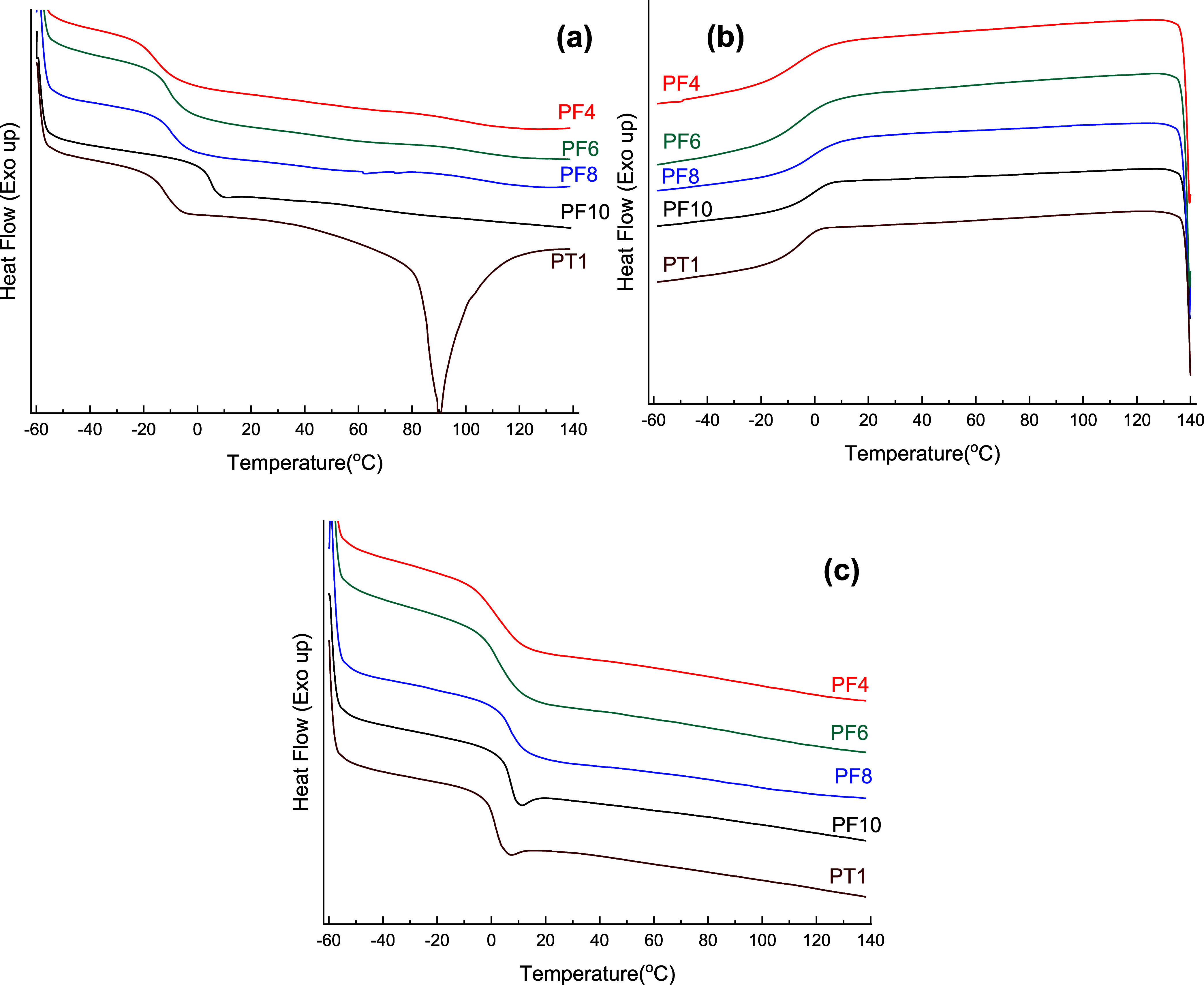
DSC thermograms of some ADMET polymers synthesized in this study: (a) first heating curve, (b) cooling scan, and (c) second heating scan.

DSC and TGA analyses were conducted to investigate the thermal properties of these novel polymers. For the furan-based polymers, the first heating scan of DSC showed a glass transition temperature (*T*_g_) in the range of −16–5 °C (Table 3 and Figure 4; also see Figure S12). A less pronounced melting endotherm with a low melting enthalpy (*H*_m_) was also evident, which is indicative of the limited crystallization of these polymers upon precipitation. However, the melting temperatures recorded here are noticeably higher than those reported earlier for 2,5-bis(hydroxymethyl)furan (BHMF)-derived copolyesters.^42^ No crystallization exotherm was apparent in the cooling and second heating scans. On the second heating scan, a *T*_g_ ranging from 1 to 7 °C was seen without any melting features, demonstrating amorphous polymer characteristics. Conversely, on the first heating scan, PT1 showed a very pronounced melting endotherm compared to its furanic counterparts, with a peak melting temperature of 90 °C and *H*_m_ = 29 J/g (also see Figure S13). This reflects the improved crystallization tendency of the TAA-based polymer, probably due to the linear structure of TAA compared to DFF.

**Table 3:** DSC and TGA Analysis Data of Some ADMET Polymers

polymer	*T*_g1_^a^ (°C)	*T*_m1_^a^ (°C)	Δ*H*_m1_^a^ (J/g)	*T*_g2_^b^ (°C)	*T*_m2_^b^ (°C)	Δ*H*_m2_^b^ (J/g)	*T*_d-5%_^c^, *T*_d-50%_^c^*, T*_d-max_^c^ (°C)	residual weight^d^ (%)
PF4	–16	111	1.38	1	e		327, 435, 440	9.0
PF6	–10	120	0.61	2	e		323, 435, 440	8.2
PF8	–10	121	0.99	7	e		241, 435, 443	9.3
PF10	5	81	0.67	7	e		350, 436, 440	7.4
PT1	–12	90	29.1	2	e		350, 442, 450	5.1
PF-OH^f^	10	67	4.73	12	73	0.65	g, 427,436	10.4
PF-NH^h^	6	71	25.67	12	e		256, 435, 443	11.7

a Glass transition temperature *T*_g1_, melting temperature *T*_m1_, and melting enthalpy Δ*H*_m1_ were all evaluated following the first heating scan of the DSC thermogram.

b Glass transition temperature *T*_g2_, melting temperature *T*_m2_, and melting enthalpy Δ*H*_m2_ were measured on the second heating scan of DSC.

c Temperature at 5% weight loss (*T*_d-5%_), 50% weight loss (*T*_d-50%_), and temperature at maximum rate of weight loss (*T*_d-max_) were determined using TGA analysis conducted under an inert atmosphere.

d Residual weight of char recovered after TGA measurement performed under an inert atmosphere.

e Not detected following the second heating scan.

f Analysis results for the hydroxy functional polymer.

g Not reported due to the presence of residual moisture.

h Analysis results for the polymer modified via reductive amination.

**Figure 5:**
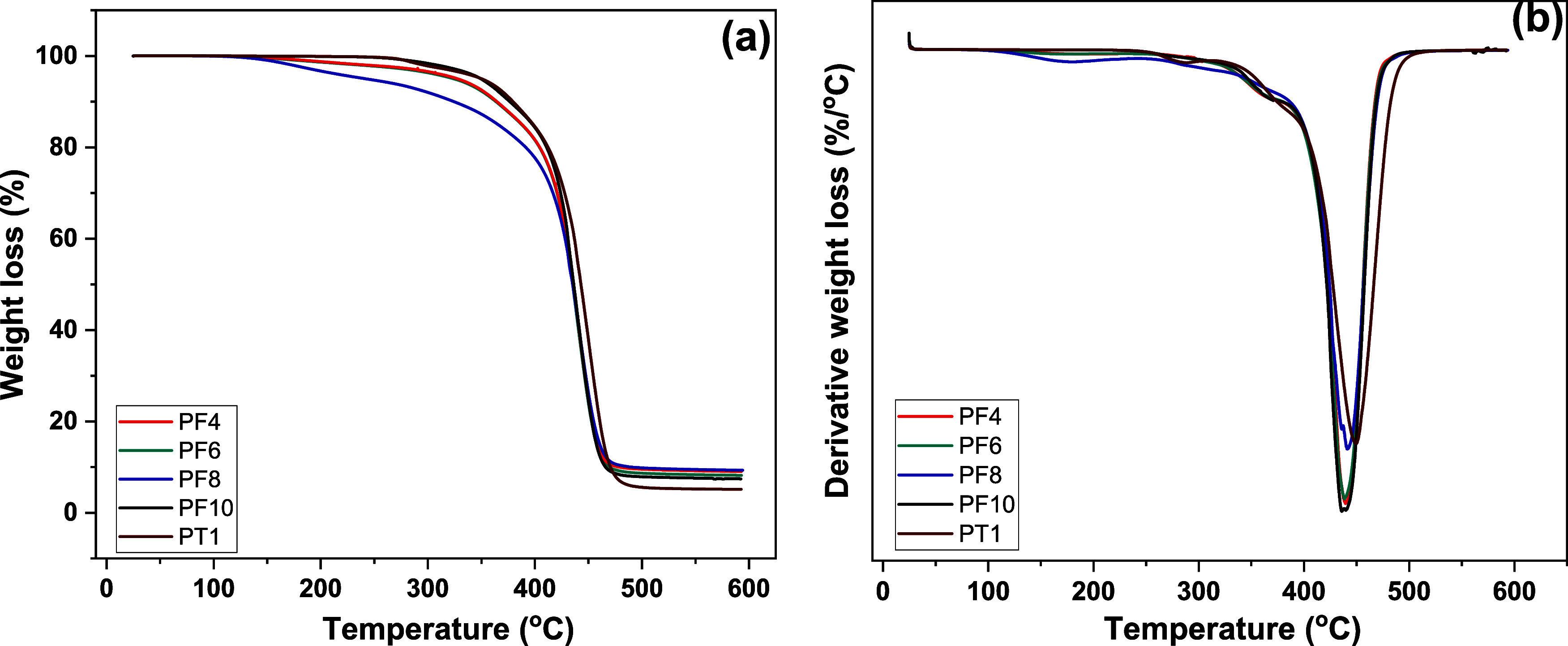
TGA curves for the ADMET polymers: (a) weight loss and (b) derivative weight loss.

TGA analysis was performed under an argon atmosphere on previously dried polymer samples. Table 3 summarizes the main outcomes from the analysis, and Figure 5 illustrates the TGA curves. Overall, the polymers synthesized in this study were found to be highly thermally stable, exhibiting onset of degradation (*T*_d-5%_) in excess of 320 °C, except for PF8, which showed signs of early degradation at 241 °C. The furanic polymers exhibited a two-step decomposition behavior with a minor degradation step initiating around 360 °C and a major decomposition featuring in the range of 450 °C. No significant difference in the thermal stability of DFF and TAA-derived polymers was apparent.

**Figure 6:**
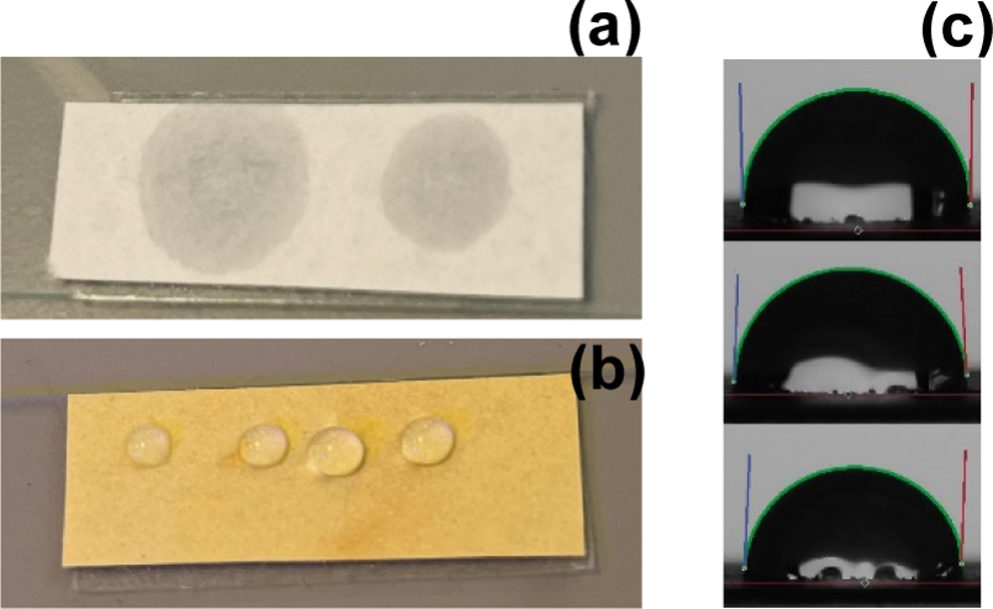
Digital pictures of a filter paper substrate used for water contact angle: (a) filter paper without polymer-coating, displaying droplet spreading and adsorption; (b) filter paper coated with a thin coating of an ADMET polymer exhibiting water hydrophobicity; and (c) images of the water droplet on the coated paper substrate used for contact angle measurements.

Owing to the long hydrocarbon chain in the polymer backbone, it was of interest to investigate the surface properties, mainly the hydrophobic properties, of these polymers. When drop-coated on the surface of a laboratory filter paper in the form of a dilute solution in THF, the polymer coating showed excellent hydrophobicity (Figure 6) upon drying at room temperature. A mean water contact angle of ∼89° was recorded (Table S3). The solubility of the polymer in common organic solvents, such as THF, makes it an excellent candidate to explore as a hydrophobic-coating on various substrates.

### Postpolymerization Modifications

**Figure 7:**
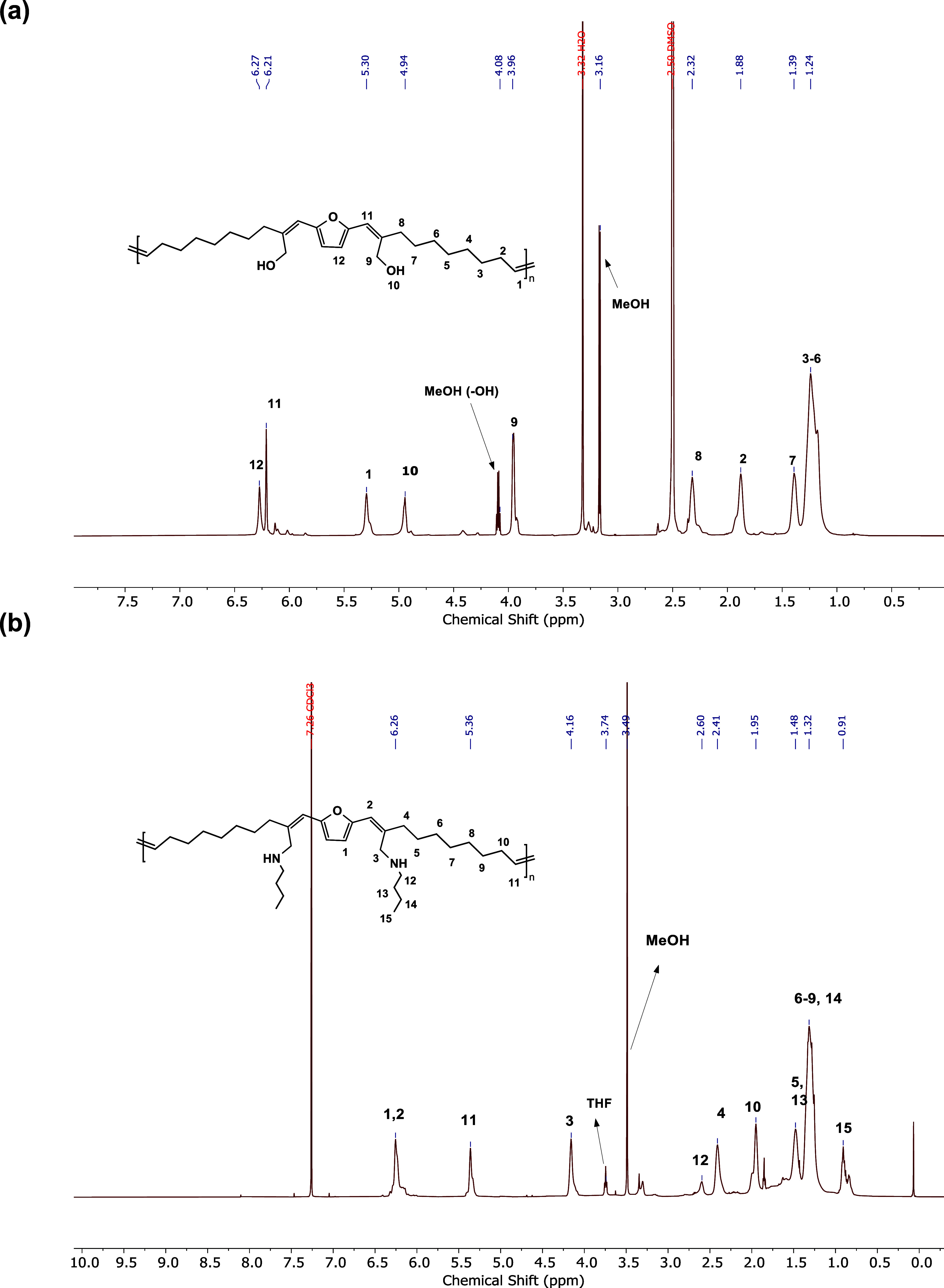
^1^H NMR spectrum of modified AMDET polymers: (a) hydroxy functional polymer (PF-OH) and (b) secondary amine functional polymer (PF-NH).

**Scheme 4:**
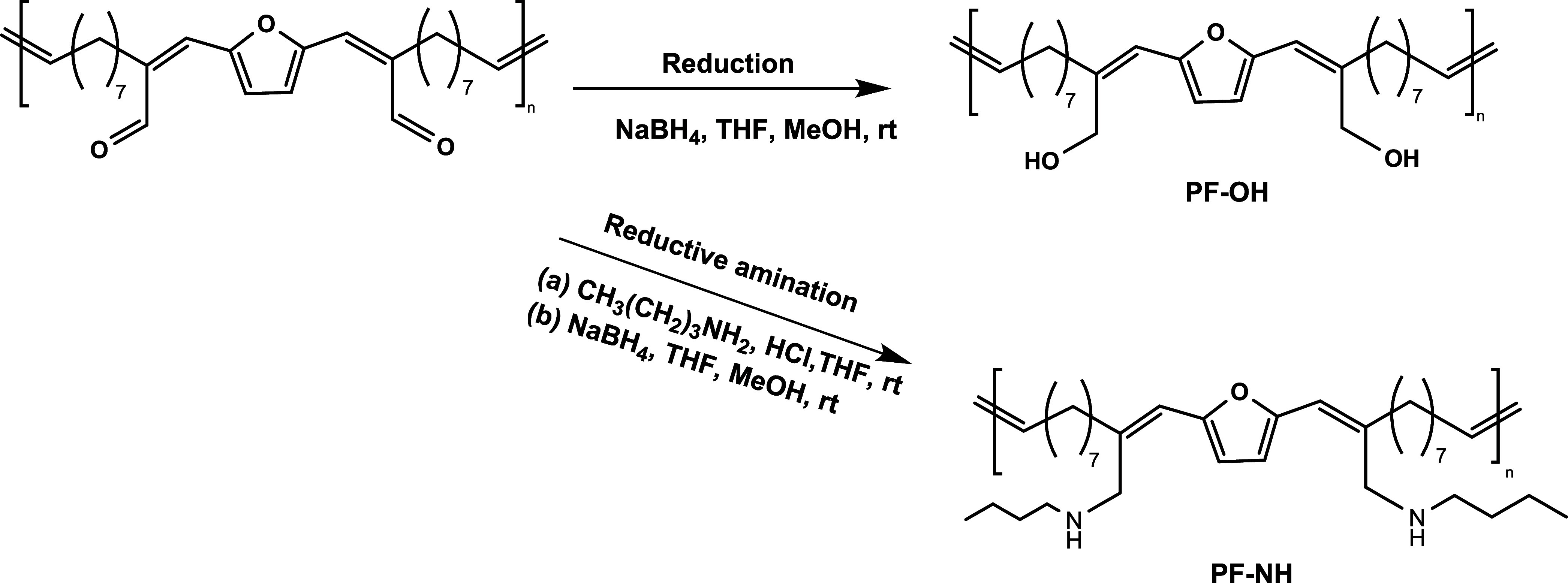
Modification Pathways Adopted for ADMET Polymers

As outlined in Figure 2, various postpolymerization structural modifications can be carried out on the newly synthesized ADMET polymers, consequently providing prospects to modify the polymer properties and design materials with targeted properties. We envisaged exploiting the aldehyde functionality on the polymer backbone for further chemical modifications. First, the aldehyde group was reduced to a hydroxy functionality using an excess of NaBH_4_ (Scheme 4). The polymer was isolated in quantitative yields after precipitation in methanol. The reduced polymer was free from any discoloration, which was previously observed in the case of unmodified ADMET polymers. The hydroxy functional polymer (PF-OH) was only partially soluble in THF and CDCl_3_, which was expected due to the development of interchain hydrogen bonding, leading to a more ordered structure. Therefore, NMR analysis was performed using DMSO-*d*_6_, in which the polymer was fully dissolved with mild heating. ^1^H NMR showed a complete reduction of the aldehyde groups, as confirmed by the disappearance of the aldehyde protons at 9.50 ppm and the emergence of two new signals at 4.02 and 5.01 ppm attributed to methylene adjacent to the conjugated alkene and hydroxyl protons, respectively (Figure 7a).

Next, we realized the reductive amination of the aldehyde group using butylamine (BA) as a model amine compound, followed by NaBH_4_ reduction, which led to a secondary amine functional polymer (PF-NH) (Scheme 4). The structural modification was confirmed by ^1^H NMR analysis conducted on a purified polymer displaying complete disappearance of aldehyde signals and appearance of new chemical shifts at 4.1 and 0.90 ppm regions assigned to the methylene group between the conjugated alkene and the secondary amino group, and methyl group of BA (H3 and H15, respectively, Figure 7b).

These results were also corroborated by FTIR analysis that showed the characteristic features of the O–H stretch (3295 cm^–1^) and N–H stretch (3317 cm^–1^) for the corresponding modified polymers, in addition to the disappearance of the C=O signals at 1673 cm^–1^ (Figure S15). Further to the solubility differences in organic solvents that was primarily observed for the hydroxy functional polymer, both modified polymers showed a noticeable difference in their thermal properties. During the first heating scan of the DSC analysis, relatively more pronounced melting endotherms were evident, exhibiting crystalline melting temperatures around 67 °C (*H*_m_ = 4.72 J/g) and 71 °C (*H*_m_ = 25.67 J/g) for the hydroxy and amine functional polymers, respectively (Table 3; also see Figure S14). This behavior can be ascribed to the interchain hydrogen bonding development, leading to the improved crystallization of the polymer. The PF-NH polymer also displayed a strong exothermic event around 117 °C on the first heating scan (Figure S14a). Detailed investigation into this phenomenon was beyond the scope of this work. However, a plausible explanation could be the residual acid-catalyzed hydroamination reaction of secondary amine and alkene functionalities, instigating the interchain cross-linking in the melt phase.^47^

The facile modification strategies presented in this work demonstrate the utility by which the polymer structure can be modified to introduce different functional groups to tailor the properties. In addition, other modification routes, such as the selective or partial hydrogenation of internal alkenes and the furan ring, and thiol-ene chemistry, can provide polymers with interesting properties and will be the subject of future investigations.^36,37,48^

### Photoactive Properties

**Figure 8:**
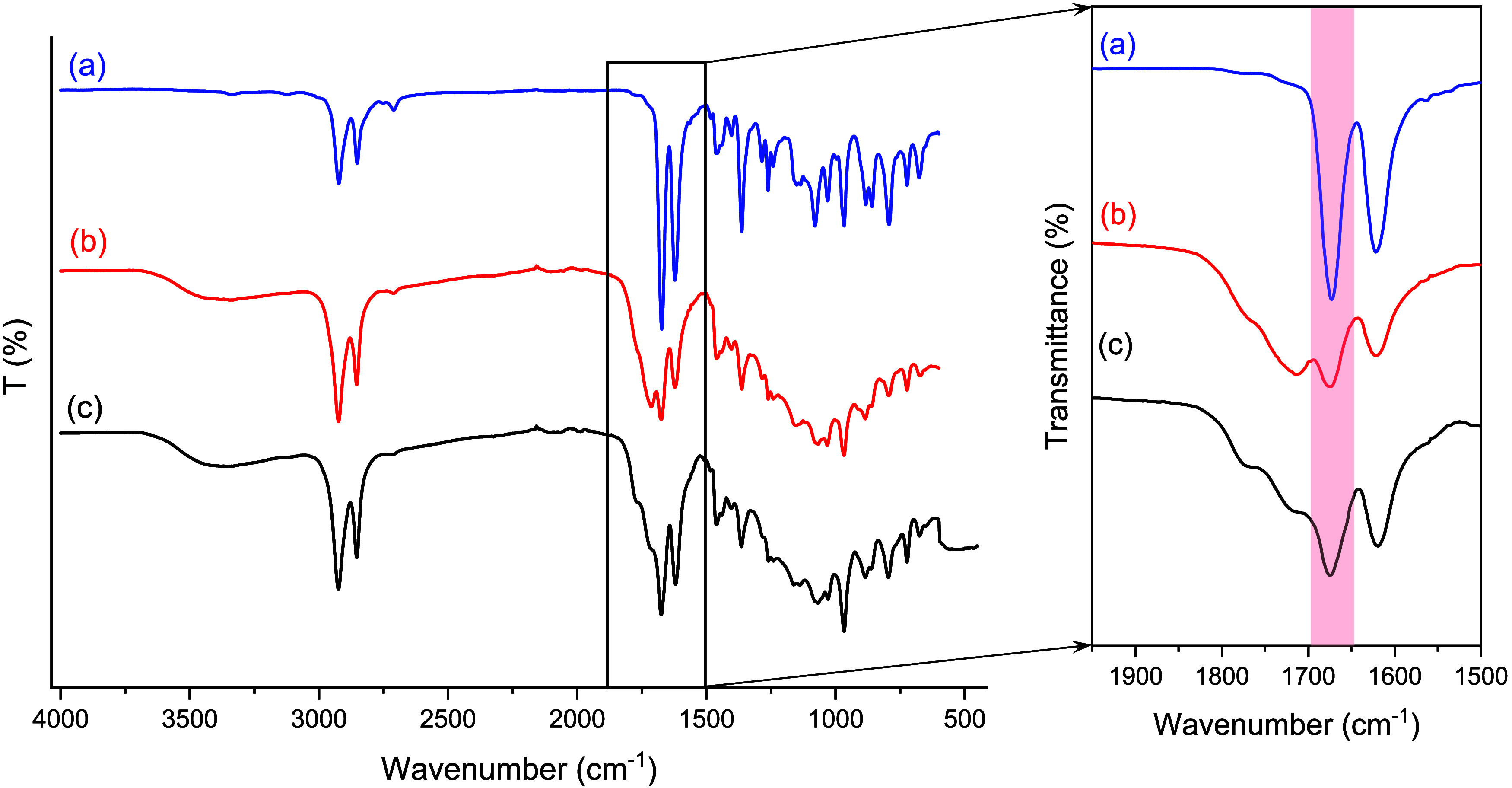
FTIR spectroscopy of ADMET polymers: (a) as synthesized, (b) after UV irradiation for 44 h, and (c) after storage in visible light at room temperature for 60 days.

Earlier literature reports have highlighted the photoactive properties of furan-based conjugated systems.^27,49^ [2 + 2] cycloaddition was the prevalent mechanism leading to cross-linking upon exposure to UV light in the absence of a photoinitiator. A preliminary investigation of the photoactivity of the current polymer system was undertaken. When a dried film (∼0.40 mm thickness) of ADMET polymer (PF8), prepared by solvent casting using THF as a solvent, was irradiated with UV light, a noticeable increase in the stiffness of the film was evident. Moreover, the irradiated sample showed limited solubility in THF and CHCl_3_; instead, an insoluble gel was obtained, which was challenging to characterize by NMR spectroscopy. FTIR analysis was conducted on the “as synthesized” polymer (without UV irradiation) and was compared with a UV cross-linked film (Figures 8 and S16). A decrease in the intensity of the band at 1691 cm^–1^ assigned to the exofuran C=C bonds and part shifting of the –C=O carbonyl signals from 1673 to 1715 cm^–1^ indicated a reduction in the conjugation after UV irradiation.^27^ Interestingly, the polymer film upon storage in visible light at ambient conditions for an extended period (60 days) revealed similar cross-linking features (Figure 8c), consequently opening the prospect of visible-light cross-linking of the polymer. This behavior was expected, given the low *T*_g_ and predominantly amorphous nature of the polymer can render greater mobility of the polymer chains in the amorphous phase at room temperature.

**Figure 9:**
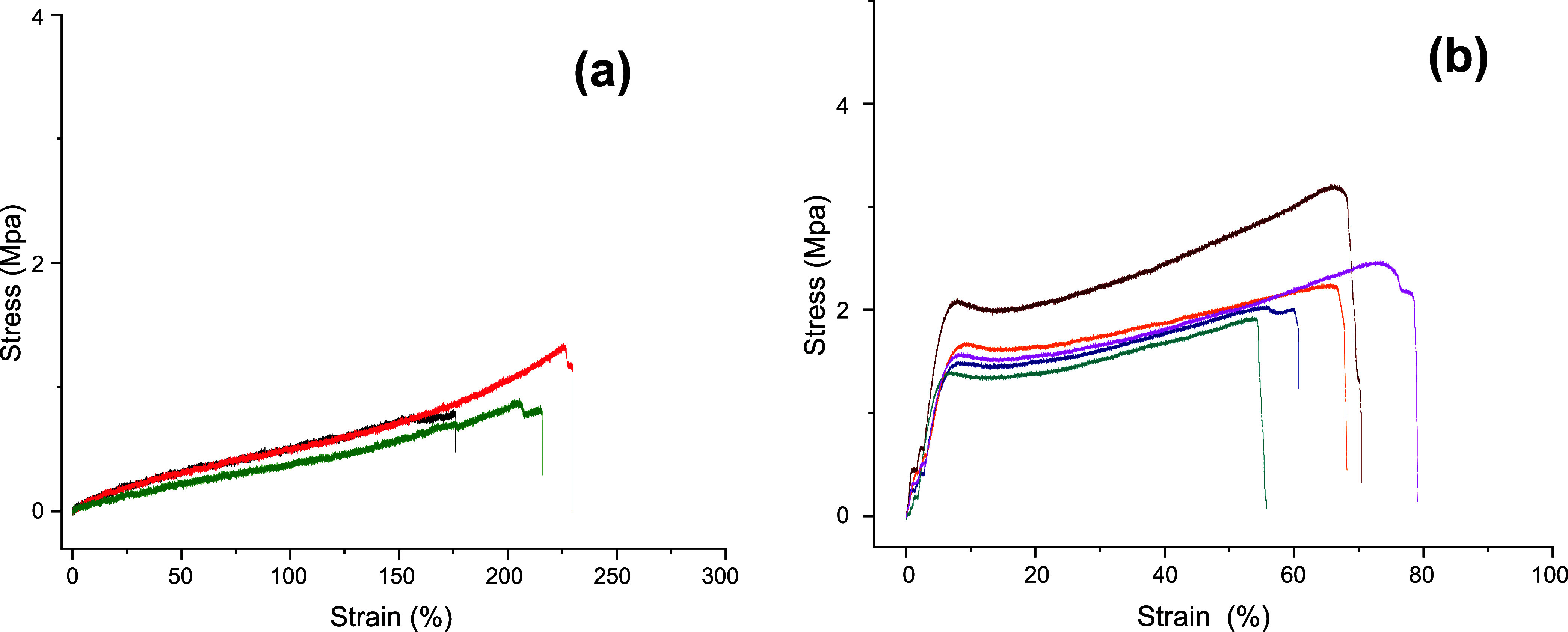
Stress–strain curves for the uniaxial tensile test conducted on the (a) as synthesized polymer and (b) polymer subjected to visible light upon storage at ambient conditions for 60 days.

The effect of cross-linking on the mechanical properties of the polymer films was also apparent once solvent-casted films were subjected to uniaxial tensile testing (Table 4 and Figure 9). The as synthesized polymer samples showed a flexible behavior with high elongation at break (206%) accompanied by a low modulus (0.81 MPa) and tensile strength (0.92 MPa). In comparison, the visible-light cross-linked polymer exhibited stiffer behavior and relatively lower elongation at break (65%); however, considerably higher modulus of 29.40 MPa and tensile strength of 2.33 MPa were recorded.

**Table 4:** Uniaxial Tensile Testing of ADMET Polymer PF8

polymer	*E*_y_^a^ (MPa)	σ_b_^b^ (MPa)	ε_b_^c^ (%)
as synthesized	0.81 ± 0.22	0.95 ± 0.28	206 ± 28
stored in visible light (60 days)	29.40 ± 5.7	2.33 ± 0.44	65 ± 8.03

a Elastic modulus.

b Tensile strength at break.

c Elongation at break.

## Conclusions

In the present work, a facile, scalable, and green approach toward the synthesis of a novel, fully renewable, and multifunctional α,ω-diene monomer (**3**) has been reported via cross-aldol condensation of DFF (**1**) and UA (**2**) in the presence of a base catalyst under mild reaction conditions. The effect of varying the reaction solvent and base catalyst on the product yield was also investigated. Excellent results were obtained when the reaction was conducted in methanol using NaOH as a base, giving monomer **3** in 78% yield within 1.5 h at room temperature. From the heterogeneous catalysts evaluated, CaO gave the desired product in 44% yield within 5 h of reaction at 40 °C. The furan-based α,ω-diene monomer was subjected to acyclic diene metathesis (ADMET) polymerization in solvent-free conditions using two metathesis catalysts—the Grubb’s second generation (G-II) and Hoveyda–Grubb’s second generation (HG-II). Once the reaction conditions were optimized using the G-II catalyst, a polymer having an *M*_n_ of 20.7 kg/mol was successfully produced at 90 °C using 0.5 mol % catalyst. For HG-II, a higher molecular weight polymer (*M*_n_ = 31 kg/mol) was obtained using a similar catalyst loading at a lower temperature (80 °C). Polymer characterization revealed the amorphous nature of the polymers having *T*_g_ in the range of −16–5 °C, possessing considerably high thermal stability (*T*_dmax_ ∼ 440 °C).

To demonstrate the potential of postpolymerization modifications, the AMDET polymer was subsequently modified by the reduction and reductive amination of the aldehyde groups, yielding hydroxyl and secondary amine functional polymers, respectively. The effect of the modifications on the structure and properties was investigated by various analytical techniques. DSC analysis of the modified polymers revealed an enhanced crystallization behavior that was attributed to hydrogen bonding interactions rendering more ordered morphology. The synthesized polymer also exhibited photoactivity toward UV and visible light, leading to a cross-linked structure having limited solubility in THF and CHCl_3_. The visible-light cross-linked polymer displayed a 35-fold increase in the tensile modulus at the expense of elongation.

Overall, this work highlights a facile and environmentally friendly route to access a novel furan-based α,ω-diene monomer that is amenable to a wide range of chemical transformations and end-use applications, with the added advantage of being fully renewable and sustainable.

## Supporting Information Available

Experimental details and general synthesis procedures, characterization methods, green metrics calculations, figures related to NMR spectroscopy and mass spectrometry analyses, GPC traces of polymers, DSC thermograms of polymers, and FTIR-ATR spectra overlays (PDF)
