# Supplementary material for: Facile Synthesis of a Novel Furanic Monomer and Its ADMET Polymerization toward Fully Renewable Functional Polymers
Source: ACS Sustain Chem Eng. 2024 Aug 29;12(37):13798–809. doi: 10.1021/acssuschemeng.4c03498 (PMC11409216; doi:10.1021/acssuschemeng.4c03498)
Supplement: Supplementary file 1 — sc4c03498_si_001.pdf [file sc4c03498_si_001.pdf]

Supporting Information

Facile synthesis of a novel furanic monomer and its ADMET polymerization towards fully renewable functional polymers

Muhammad Kamran,* [a,b] Andrew Kay, [a,b] Matthew G Davidson,* [a,b]

[a] Institute for sustainability, University of Bath, Claverton Down, BA2 7AY UK.

[b] Department of Chemistry, University of Bath, Claverton Down, BA2 7AY, UK.

*Email: m.kamran@bath.ac.uk, m.g.davidson@bath.ac.uk

Number of pages: 26

Number of figures: 16

Number of schemes: 2

Number of tables: 3

Contents

1 Experimental....3
1.1 Materials....3
1.2 Synthesis of 2,5- diformylfuran (DFF) 3
1.3 Synthesis of α,ω-diene monomer 3....3
1.3.1 Small scale synthesis....3
1.3.2 Multi-gram scale synthesis 4
1.4 General procedure for ADMET polymerization of monomer 3 4
1.5 Terephthalaldehyde (TAA) based monomer 5 synthesis....5
1.6 Procedure for ADMET polymerization of monomer 5....6
1.7 General procedures for post-polymerization modifications 6
1.7.1 Aldehyde reduction 6
1.7.2 Reductive amination....7
1.8 Procedure for polymer film preparation for photoactivity and tensile tests 7
2 Characterization 7
3 Green metrics calculations for monomer 3 synthesis via cross-aldol condensation....8
3.1 Green metrics comparison 10
4 NMR spectroscopy analyses....11
4.1 2,5-diformylfuran (DFF) 11
4.2 Furan-based α,ω-diene monomer 3 12
4.2.1 2D NMR spectra for the furan-based α,ω-diene monomer 3....13
4.3 Furan-based ADMET polymer....15
4.4 Double bond isomerization 16
4.5 NMR spectroscopy of TAA-based monomer 5....17
4.6 ADMET polymer PT1....18
5 Mass spectrometry....19
5.1 Mass spectrometry of α,ω-diene monomer 3....19
5.2 Mass spectrometry of TAA-based α,ω-diene monomer 5....19
6 GPC traces of ADMET polymers....20
7 DSC analyses curves....21
7.1 DSC analysis of fully renewable furan-based ADMET polymers 21
7.2 DSC of terephthalaldehyde (TAA) – based ADMET polymer (PT1) 22
7.3 DSC thermograms of Furan-based ADMET polymer after modification....23
7.4 Polymer hydrophobicity and water contact angle on coated filter paper....24
8 FTIR spectroscopy analyses 25
8.1 ADMET polymer post-polymerization modification 25
8.2 Photoactivity analysis by FTIR spectroscopy....25
9 References....26

1 Experimental

1.1 Materials

All chemicals were obtained from commercial sources and used without any purification unless stated otherwise. 5-hydroxymethylfurfural (HMF, 99%) was supplied by Fluorochem UK. Manganese(IV) oxide (MnO_2, 88% electrolytically precipitated), 10-undecenal (UA, 97%) and MgO were purchased from Thermo Scientific UK. CaO was acquired from Alfa Aesar. $MgSO_4$ was obtained from Fisher. Grubbs 2nd generation (M204), Hoveyda-Grubbs 2nd generation (M720) catalysts, Ethyl vinyl ether (EVE), butylamine (BA, 99.5%), sodium borohydride ($NaBH_4$, 98%) K_2CO_3 and NaOH were all acquired from Merck. All solvents were of analytical grade and used as received. Both heterogenous catalysts (MgO and CaO) were activated in the presence of air at a furnace temperature of 500 °C for 5 hours before use.

1.2 Synthesis of 2,5- diformylfuran (DFF)

2,5- diformylfuran **1** (DFF) was successfully synthesized in excellent yields starting from HMF using manganese(IV) oxide as the oxidant following a slight modification of the method already described elsewhere.[1] Briefly, in an oven-dried 250 mL round flask with a magnetic stirrer, HMF (5230 mg, 41.471 mmol, 1 equiv) was dissolved in 55 ml DCM under vigorous string. MnO_2 (10060 mg, 115.70 mmol, 2.79 equiv) was added in portions and the mixture was refluxed under argon flow for 24 hours. The reaction mixture was subsequently diluted with EtOAc once cooled down to room temperature, followed by filtration using a Büchner funnel to remove the MnO_2 particles. The MnO_2 residue was washed with fresh EtOAc at least five times to recover the residual DFF. The flask was subjected to rotary evaporation to recover the crude DFF as yellow solid. Crude DFF was purified by recrystallization from toluene or by column chromatography (silica gel, DCM: MeOH 100:0 to 97:3) to give DFF as a white crystalline solid. Yield 78%, melting point = 112 °C (DSC).

TLC (DCM: MeOH= 90:10) R_f= 0.62

^{1}H NMR (500 MHz, $CDCl_3$, δ in ppm): 9.86 (2H, H-1), 7.27 (2H, H-2).

^{13}C {^{1}H} NMR (125 MHz, $CDCl_3$, δ in ppm): 179.3 (C-a), 154.4 (C-b), 119.3 (C-c).

1.3 Synthesis of α,ω-diene monomer 3

1.3.1 Small scale synthesis

The synthesis procedure for the run was performed at room temperature using methanol as a solvent and NaOH as a base catalyst (Table 1, run 1), which will be described here as a representative example.

DFF **1** (106.8 mg, 0.86 mmol, 1.0 equiv.) was carefully weighed in a glass vial with a magnetic stirrer before adding 5 mL methanol. The mixture was vigorously stirred at room temperature until DFF was

completely dissolved. Subsequently, 0.3 mL of 1 M aqueous NaOH was injected with stirring for another 5 minutes, followed by 10-undecenal **2** (0.33 mL, 2.01 equiv.) addition. The reaction was stirred for 90 minutes in total, after which a sample was taken for quantitative ^{1}H NMR. The rest of the crude reaction mixture was transferred to a separating funnel and extracted with n-hexane (15 mL x 3). The organic layers were combined and dried over anhydrous $MgSO_4$ and filtered. After removal of the solvent, the crude monomer was obtained as a yellow oil, which was purified *via* column chromatography (silica gel, *n*-hexane: ethyl acetate 100:0 to 70:30) to give a bright pale-yellow oil. Yield 68%. Due to the toxicological concerns (dermal, reproductive, acuatic) associated with n-hexane, readers are encouraged to test low-toxicity biomass-derived solvents, for instance, ethyl-lactate, ɤ-valerolactone and limonene to replace n-hexane.

For the experiment conducted in the absence of solvent, methanolic NaOH (1 M) was used as a base catalyst. For K_2CO_3 catalyzed reaction, 0.3 mL of a 1 M aqueous solution was employed. For heterogenous bases (CaO and MgO), 27 mg of each catalyst was employed.

1.3.2 Multi-gram scale synthesis

In an oven-dried round bottom flask with a magnetic stirrer, DFF **2** (1820 mg, 14.67 mmol, 1.0 equiv) and 40 mL methanol were stirred until complete dissolution. Subsequently, 1.8 mL NaOH (3 M) was injected, and the solution was stirred for another 5 minutes before adding 10-undecenal **1** (4960 mg, 29.48 mmol, 2.01 equiv). The reaction was continued at room temperature during which the progress of the reaction was monitored *via* TLC using *n*-hexane: ethyl acetate 80:20 as eluent. TLC showed complete conversion of DFF in an hour. The reaction was run for another 30 minutes (total 90 minutes). 1 M HCl was then added to adjust the pH to 7-8, followed by extraction of the aqueous (methanol) phase with *n*-hexane (50 mL x 3). The organic layers were combined and dried over anhydrous $MgSO_4$ and filtered. After removing the solvent in the rotovap, the crude aldol monomer was obtained as a yellow oil. The crude product was purified *via* column chromatography (silica gel, *n*-hexane: ethyl acetate 100:0 to 70:30) to give a bright pale-yellow oil. Yield 78%.

TLC (*n*-hexane: ethyl acetate = 80:20), R_f= 0.30

^{1}H NMR (500 MHz, $CDCl_3$, δ in ppm): 9.49 (2H, H-1), 6.94 (2H, H-3), 6.88 (2H, H-2), 5.76 (2H, H-11), 4.91 (4H, H-12), 2.65 (4H, H-4), 2.01 (4H, H-10), 1.45 (4H, H-5), 1.22-1.38 (16H, H-6 to H-9).

^{13}C {^{1}H} NMR (125 MHz, $CDCl_3$, δ in ppm): 193.6 (C-a), 153.3 (C-c), 141.9 (C-e), 139.0 (C-m), 133.6 (C-d), 118.6 (C- b), 114.2 (C-n), 33.7 (C-l), 29.6 – 28.8 (C-h to C-k), 28.3 (C-g), 25.0 (C-f).

1.4 General procedure for ADMET polymerization of monomer 3

The purified monomer **3** (1.80 g, 1.0 equiv) was carefully weighted into an oven-dried two-neck round bottom flask. A pre-determined amount of Grubbs 2nd generation (G-II) or Hoveyda-Grubbs 2nd

generation (HG-II) catalyst was subsequently added to the monomer. A vacuum-tight magnetic stirrer coupler (Buddeberg, Germany) fitted with a PTFE-coated steel stirring rod was inserted for the mechanical stirring. The flask was connected to an overhead stirrer and vacuum/argon line. Initially, the reaction contents were flushed with argon for ca. 15 minutes and degassed three times. Stirring was then initiated and the reaction was gradually heated to the desired temperature under a dynamic vacuum (1-2 mbar). The reaction was stopped by disconnecting the vacuum and removing the flask from the heating block. Once the polymer cooled down to room temperature, THF (5-7 mL) was added to dissolve the polymer, followed by the addition of Ethyl vinyl ether (1.5 mL) with stirring for 30 minutes to quench the reaction. The purification of polymers from the residual monomer/oligomers was carried out by precipitation into cold methanol (60 mL). The purified polymer was isolated following centrifugation (3800 rpm, 8 minutes). The isolated polymer was washed with fresh methanol three times and dried on the vacuum line. Further analyses were conducted on the purified polymer samples. For reaction conversion estimation by ^{1}H NMR, a crude polymer sample was taken before the purification step.

^{1}H NMR (500 MHz, $CDCl_3$, δ in ppm): 9.50 (2H, H-1), 6.95 (2H, H-3), 6.88 (2H, H-2), 5.34 (2H, H-13), 2.65 (4H, H-4), 1.93 (4H, H-10), 1.45 (4H, H-5), 1.36-1.25 (16H, H-6 to H-9).

^{13}C {^{1}H} NMR (125 MHz, $CDCl_3$, δ in ppm): 194.0 (C-a), 153.4 (C-c), 142.2 (C-e), 133.9 (C-d), 130.5 (C-m), 118.7 (C- b), 32.7 (C-l), 29.9 – 29.2 (C-h to C-k), 28.5 (C-g), 25.3 (C-f).

1.5 Terephthalaldehyde (TAA) based monomer 5 synthesis

In an oven-dried round bottom flask with a magnetic stirrer, terephthalaldehyde (**4**, TAA) (2128 mg, 15.87 mmol, 1 equiv.) was dissolved in 25 mL methanol. After complete dissolution, 1 mL NaOH (3 M) was injected and stirred for another 5 minutes, followed by the addition of 10-undecenal **2** (5610 mg, 2.01 equiv.). While precipitate formation was already observed within an hour, the contents were allowed to stir for approx. 4 hours at room temperature. 10 mL fresh methanol was added to dilute the precipitates, which were allowed to stir for another hour (total 5 hours). The reaction flask was subsequently removed from the string and transferred to a fridge. After 5 days, the precipitates were filtered and washed with cold methanol 5 times (25 mL each). The isolated product obtained as a white crystalline powder was dried in the vacuum oven overnight and used without any further purification. Yield 46%, melting point = 61°C (DSC).

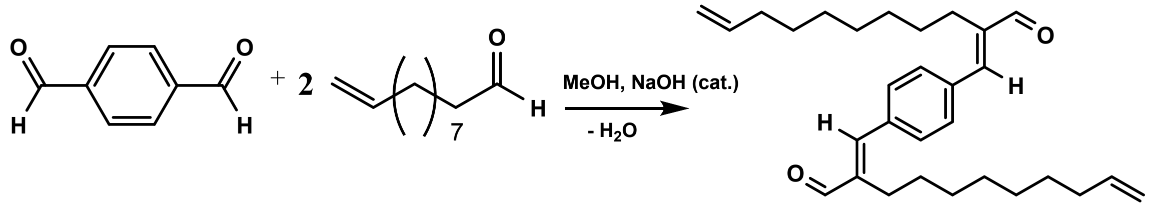

4 2 5

Scheme S1. Synthesis scheme representing the α,ω-diene monomer (**5**) from terephthalaldehyde (TAA, **4**) by aldol-condensation reaction.

^{1}H NMR 500 MHz, $CDCl_3$, δ in ppm: 9.57 (2H, H-3), 7.57 (4H, H-1), 7.21 (2H, H-2), 5.79 (2H, H-11), 4.94 (4H, H-12), 2.54 (4H, H-4), 2.03 (4H, H-10), 1.51 (4H, H-5), 1.37-1.31 (16H, H-6 to H-9).

^{13}C {^{1}H} NMR (125 MHz, $CDCl_3$, δ in ppm): 195.4 (C-a), 148.3 (C-c), 144.3 (C-b), 139.1 (C-m), 136.0 (C-d), 130.0 (C-e), 114.2 (C- n), 33.7 (C-l), 29.8 – 28.9 (C-h to C-k), 28.3 (C-g), 24.9 (C-f).

1.6 Procedure for ADMET polymerization of monomer 5

Dried monomer **5** (1487 mg, 3.42 mmol, 1 equiv.) was premixed with HG-II catalyst (11 mg, 0.017 mmol) and transferred to an oven-dried two-neck round bottom flask fitted with an overhead stirrer and vacuum-tight stirring coupler. The reaction contents were flushed with argon and degassed under vacuum three times before they were heated under an argon atmosphere to 70 °C to allow the monomer to melt completely (approx.10 minutes). Overhead mechanical stirring was then initiated, and the temperature was gradually increased to 90°C under dynamic vacuum pressure. The remaining reaction procedure and the isolation and purification steps were similar to those applied to the furanic monomer **3** and have already been described in section 1.4. Yield 85%.

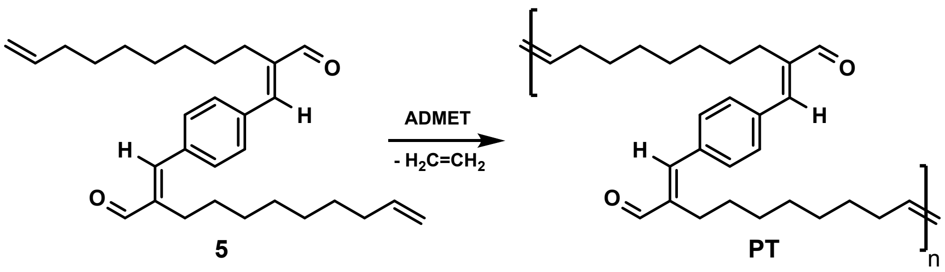

Scheme S2. ADMET polymerisation of TAA-based monomer **5**.

^{1}H NMR 500 MHz, $CDCl_3$, δ in ppm: 9.56 (2H, H-3), 7.55 (4H, H-1) ,7.21 (2H, H-2), 5.36 (2H, H-11), 2.54 (4H, H-4), 1.94 (4H, H-10), 1.49 (4H, H-5), 1.37- 1.28 (16H, H-6 to H-9).

^{13}C {^{1}H} NMR (125 MHz, $CDCl_3$, δ in ppm): 195.4 (C-a), 148.3 (C-c), 144.3 (C-b), 136.0 (C-d), 130.3 (C-m), 130.0 (C-e), 32.6 (C-l), 29.88 – 29.1 (C-h to C-k), 28.3 (C-g), 24.9 (C-f).

1.7 General procedures for post-polymerization modifications

1.7.1 Aldehyde reduction

In a 28 mL vial with a magnetic stirrer, 53.2 mg (0.109 mmol, 1 equiv., relative to polymer repeat unit) of the polymer was weighed, followed by the addition of 2.5 mL of THF. The mixture was stirred until the polymer was completely dissolved. Subsequently, an excess of $NaBH_4$ (50.0 mg, 12 equiv.) dissolved in a minimum quantity of methanol separately, was gradually added to the first vial under vigorous stirring. The contents were allowed to be stirred overnight at room temperature. The solution

was concentrated on the rotovap and precipitated dropwise into cold methanol (25 mL). A transparent polymer (PF-OH) was recovered in quantitative yield after centrifugation and fresh methanol washes.

^{1}H NMR 500 MHz, $CDCl_3$, δ in ppm: 6.27 (2H, H-12), 6.21 (2H, H-11), 5.30 (2H, H-1), 4.94 (2H, H-10), 3.96 (4H, H-9), 2.32 (4H, H-8), 1.88 (4H, H-2), 1.39 (4H, H-7), 1.20- 1.30 (16H, H-3 to H-6).

1.7.2 Reductive amination

To a solution of ADMET polymer (68.7 mg, 0.162 mmol, 1 equiv.) in 3.0 mL THF was added butylamine (35.5 mg, 3.0 equiv.) and stirred for 5 minutes. The pH of the solution of adjusted to around 5 using HCl (1 M). A distinct colour change from light brown to dark orange was observed upon acidification. Subsequently, another batch of 1.5 equiv. of butylamine was added, followed by pH adjustment. The reaction was continued for 4 hours at room temperature. $NaBH_4$ (25 mg) was directly added in the solution under vigorous stirring and left overnight for the imine reduction step. The amine-modified polymer (PF-NH) was recovered after similar purification and isolation steps described above.

^{1}H NMR 500 MHz, $CDCl_3$, δ in ppm: 6.26 (4H, H-1 To H-2), 5.36 (2H, H-11), 4.16 (4H, H-3, 2.60 (4H, H-12), 2.41 (4H, H-4), 1.95 (4H, H-10), 1.48 (8H, H-5, H-13), 1.32 (20H, H-6 to H-9, H-14), 0.91 (6H, H-15).

1.8 Procedure for polymer film preparation for photoactivity and tensile tests

Solvent casted films were prepared by carefully pouring the polymer solution (2.2 g polymer in 6 mL THF) into two separate circular Teflon moulds. Moulds were subsequently covered with a perforated foil and left in a well-ventilated fumehood until the solvent had evaporated (5 days). The films were removed from the moulds after quenching in liquid nitrogen. The isolated films were dried in a vacuum oven at 30°C for 2 hours before subjecting them to any further treatment or analysis.

The "as synthesized" film was analyzed without any further treatment. For UV irradiated sample, a UV light chamber (λ = 365 nm) from MelodySusie® (36 W UV Nail Dryer) was employed. The visible light crosslinked specimen was produced after leaving the "as synthesized" casted film in ambient light for 60 days.

2 Characterization

NMR spectra were recorded at 25 °C on 500 MHz Bruker Avance II+ instrument. All spectra were referenced to the chemical shifts associated with the deuterated solvents.

FTIR-ATR spectroscopy was performed on a PerkinElmer spectrometer two. For a single run, a total of 8 scans were recorded at a 1 cm^{-1} spectral resolution.

Differential scanning calorimetry (DSC) analysis was conducted on a TA-instrument Discovery DSC25 series instrument. All runs were performed under a nitrogen atmosphere, with a constant flow rate of 50 mL min^{-1}. For the DSC runs, 3-5 mg of the sample was weighed into the pan. In the analysis program, samples were first equilibrated at -60 °C before heating them to 140 °C to construct the first heating

scan. Samples were then cooled to -60 °C during the cooling scan and heated again to 140°C for the second heating scan. All samples were analyzed using the same heating/cooling rate of 10°C min^{-1}. Thermogravimetric analysis (TGA) was performed on a TA-instrument Discovery TGA550. 12-15 mg sample, pre-dried at 60 °C in a vacuum oven for 2 hours, was loaded into the TGA pans. In the analysis program, samples were heated from room temperature to 600 °C at 10 °C min^{-1}, under a constant flow of nitrogen. From the TGA curves, the corresponding temperatures for 5% weight loss ($T_{d\text{-}5\%}$), 50% weight loss ($T_{d\text{-}50\%}$) and temperature at a maximum rate of weight loss ($T_{d\text{-}max}$) were reported.

Gel permeation chromatography (GPC) was performed on an Agilent 1260 Infinity GPC/SEC system, equipped with a refractive index (RI) detector, two analytical columns (PLgel 5 µm MIXED-D 300 × 7.5 mm), and a guard column PLgel 5 µm MIXED Guard 50 × 7.5 mm, all maintained at 35 °C. The GPC system was calibrated using polystyrene standards. GPC-grade THF was used as a mobile phase at a flow rate of 1.0 mL min^{-1}. Samples were prepared in THF at a 2 mg mL^{-1} concentration and an injection volume of 100 µL was employed.

Uniaxial tensile testing was performed on a universal testing machine by AML instrument (model: Z10 X1200) equipped with 1 kN load cell. Tensile tests were conducted at a 2mm min^{-1} strain rate on the specimen of dimensions 35 mm x 5.5 mm x 0.35 mm, cut from the solvent-casted films. At least three replicates were tested, and average values were reported alongside the standard deviation.

Water contact angle measurements were conducted using Ossila contact angle goniometer. A laboratory filter paper (Watman, diameter: 125mm) was drop coated with a dilute solution of polymer (23 mg mL^{-1}) and left in a well-ventilated fumehood until the solvent evaporation (overnight). Contact angles for three deionized water drops placed at different locations on the coated paper were evaluated, and an average angle was reported.

3 Green metrics calculations for monomer 3 synthesis via cross-aldol condensation

$$Atom\ Economy\ (AE) = \frac{Mr\ product}{Mr\ all\ products} \times 100$$

$$= \frac{Mr\ Monomer\ 3}{(Mr\ Monomer\ 3) + 2(Mr\ H_2O)} \times 100$$

$$= \frac{424.62}{424.62 + 36.04} \times 100$$

$$= 92\%$$

$$Reaction\ Mass\ Efficiency\ (RME) = \frac{Mass\ of\ product\ C}{Mass\ of\ A + Mass\ of\ B} \times 100$$

$$= \frac{Mass\ of\ monomer\ 3}{Mass\ of\ DFF + Mass\ of\ Undencenal}$$

$$= \frac{4859}{1820 + 4960} \times 100 \ = 72\%$$

Table S1. Mass balance for the synthesis of monomer 3 at multi-gram scale.

S.No	Reagent	Mass Used (g)	Mass of waste (g)	Mass Produced (g)
1	DFF	1.82	0	
2	Undecenal	4.96	0.24	
3	MeOH	31.68	31.68	
4	NaOH	0.18	0.18	
5	Water	1.8	1.8	
6	Hexane (extraction)	99.15	99.15	
7	Column (1:4 EtOAc:Hexane)	354.60	354.60	
	Total solvent used (3+6+7)	485.43	48.54[a]	
8	Monomer 3			4.86

[a]Assuming solvent recycling results in a 10% loss as waste, while 90% is recovered and re-used

$$Process\ Mass\ Index\ (PMI) = \frac{Total\ mass\ used\ in\ synthesis}{Mass\ of\ product} \quad \text{(Including solvents)}$$

$$= \frac{1.82 + 4.96 + 31.68 + 0.18 + 1.8 + 99.15 + 354.60}{4.86}$$

$$= 101.34$$

$$Process\ Mass\ Index\ (PMI) = \frac{Total\ mass\ used\ in\ synthesis}{Mass\ of\ product} \quad \text{(Including solvent with 90\% recycling)}$$

$$= \frac{1.82 + 4.96 + 0.18 + 1.8 + 48.54}{4.86}$$

$$= 11.79$$

$$Simple\ E\ factor\ (sEF) = \frac{Total\ mass\ of\ waste}{Mass\ of\ product} \quad \text{(Excluding solvents)}$$

$$= \frac{0.24 + 0.18 + 1.8}{4.85}$$

$$= 0.46$$

$$Complete\ E\ factor\ (cEF) = \frac{Total\ mass\ of\ waste}{Mass\ of\ product} \quad \text{(Including solvent with 90\% recycling)}$$

$$= \frac{0.24 + 0.18 + 1.8 + 48.54}{4.85}$$

$$= 10.05$$

$$Carbon\ Efficiency = \frac{6 + 2(11)}{28} \times 100 = 100\%$$

$$Atom\ Efficiency = \frac{AE\ \times \%\ yield}{100}$$
$$= \frac{92\%\ \times 78\%}{100}$$
$$= 72\ \%$$

3.1 Green metrics comparison

Table S2. Green metrics comparison for various synthesis approaches and current work.

Green metrics	Ref. [2]	Ref. [3]	Ref. [4]	This work
**Atom Economy**	63%	87%	86%	92%
**Atom Efficiency**	31%	67%	51%	72%
**Simple E-factor**	4.7	0.69	1.58	0.46
**Reaction Mass Efficiency**	21%	55%	43%	72%

4 NMR spectroscopy analyses

4.1 2,5-diformylfuran (DFF)

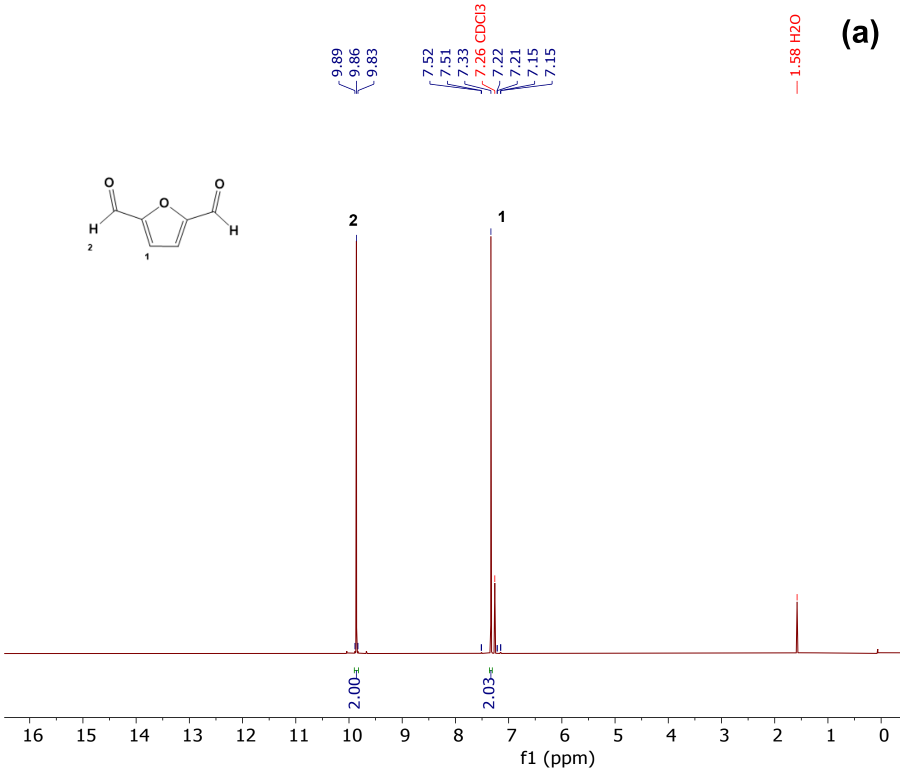

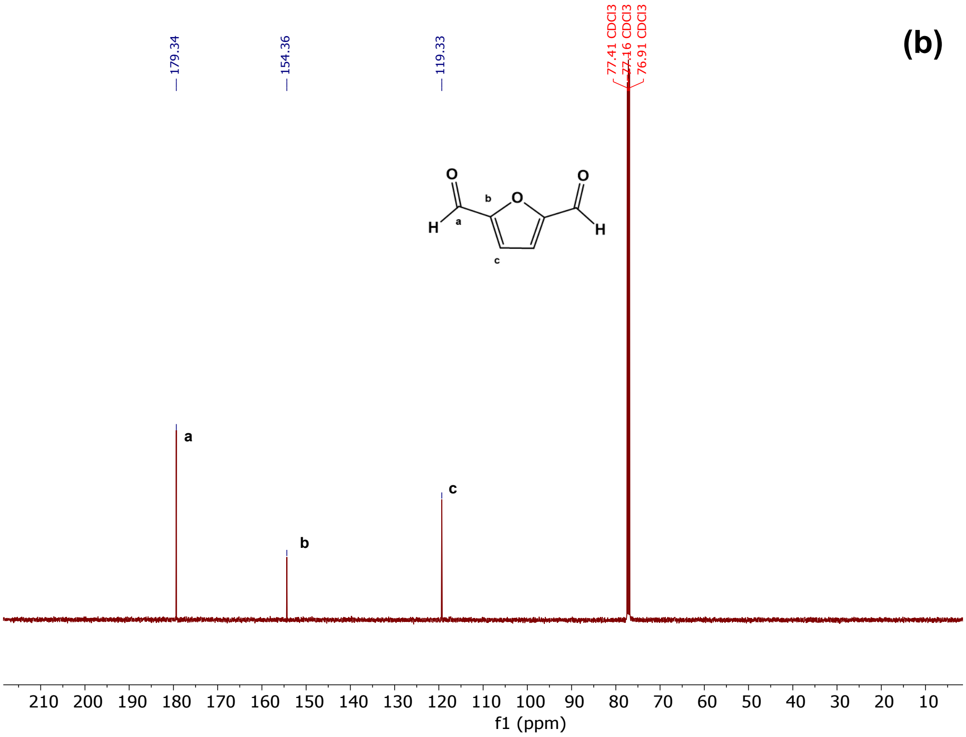

Figure S1. NMR spectroscopy of 2,5-diformylfuran (DFF) with assignments performed in $CDCl_3$ at 25 °C; **(a)** ^1H NMR **(b)** {^1H} ^{13}C NMR.

4.2 Furan-based α,ω-diene monomer 3

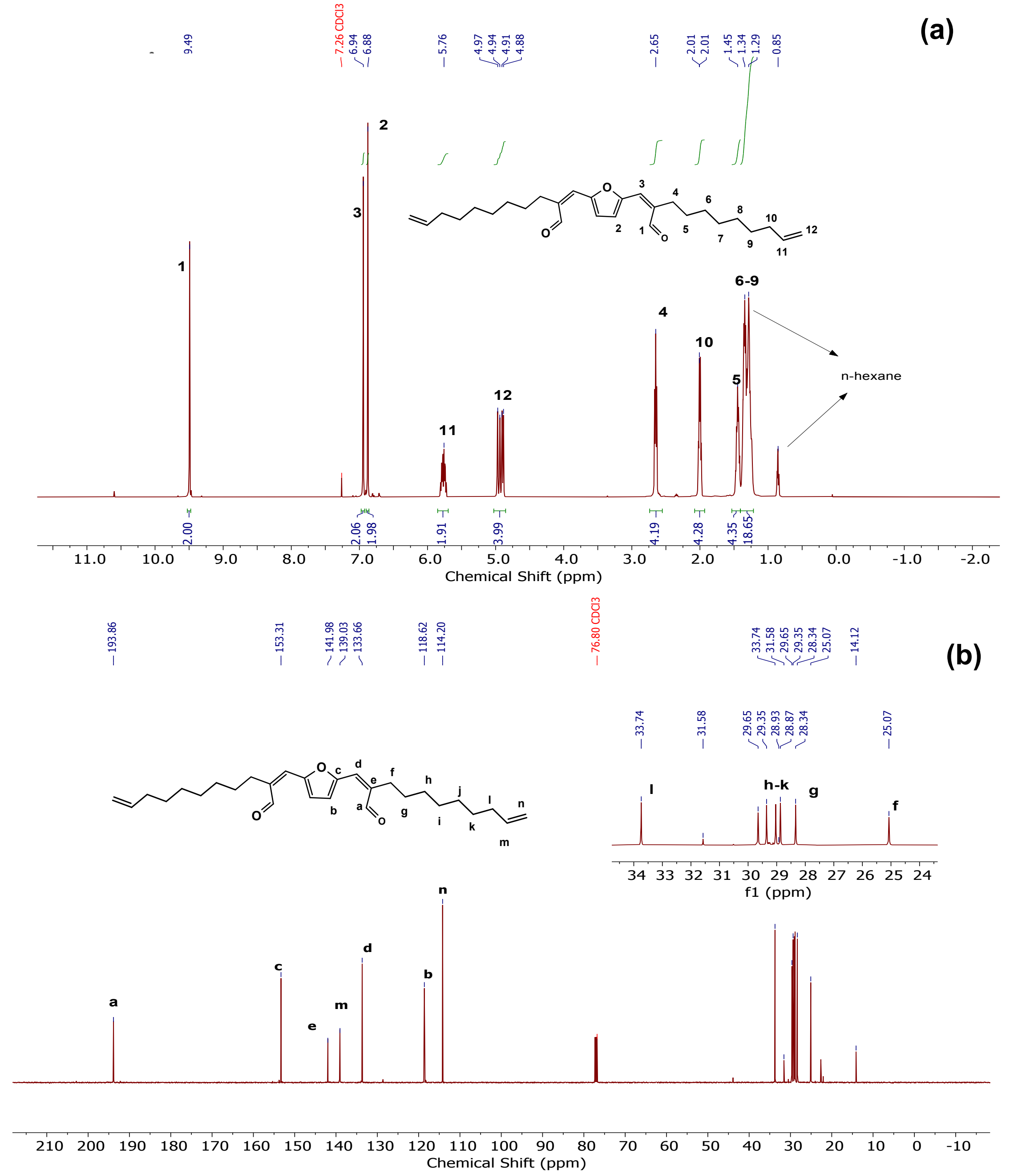

Figure S2. NMR analyses of furan-based α,ω-diene monomer depicting the corresponding assignments conducted in $CDCl_3$ at 25°C; **(a)** ^1H NMR **(b)** {^1H} ^{13}C NMR.

4.2.1 2D NMR spectra for the furan-based α,ω-diene monomer 3

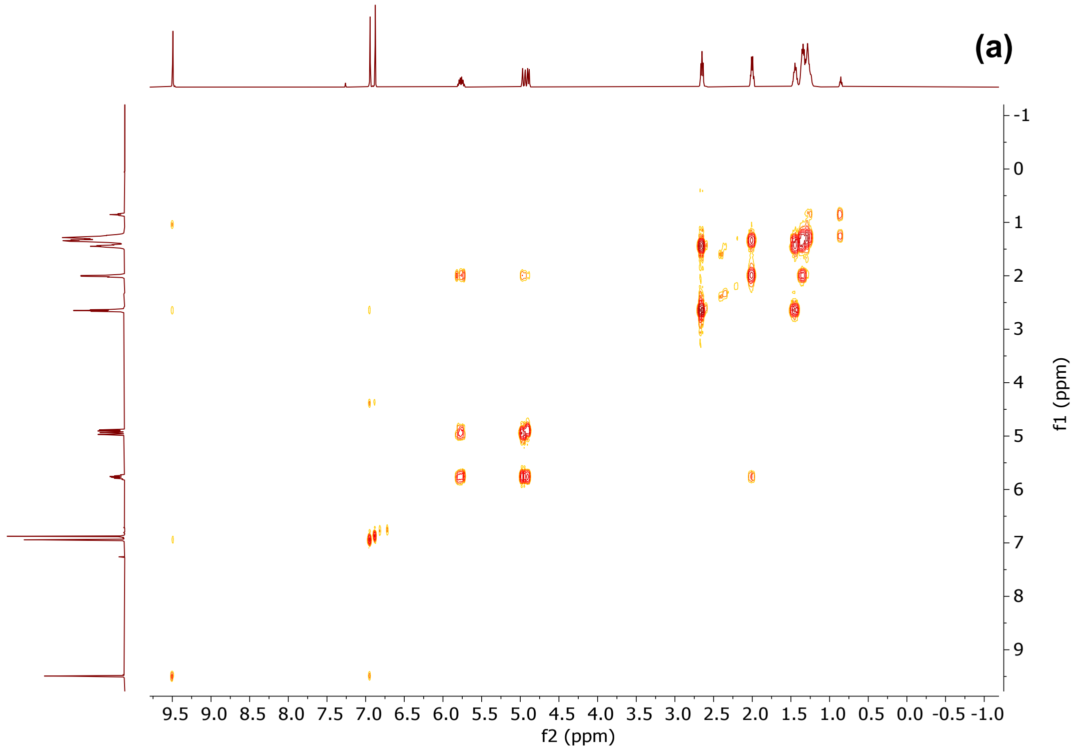

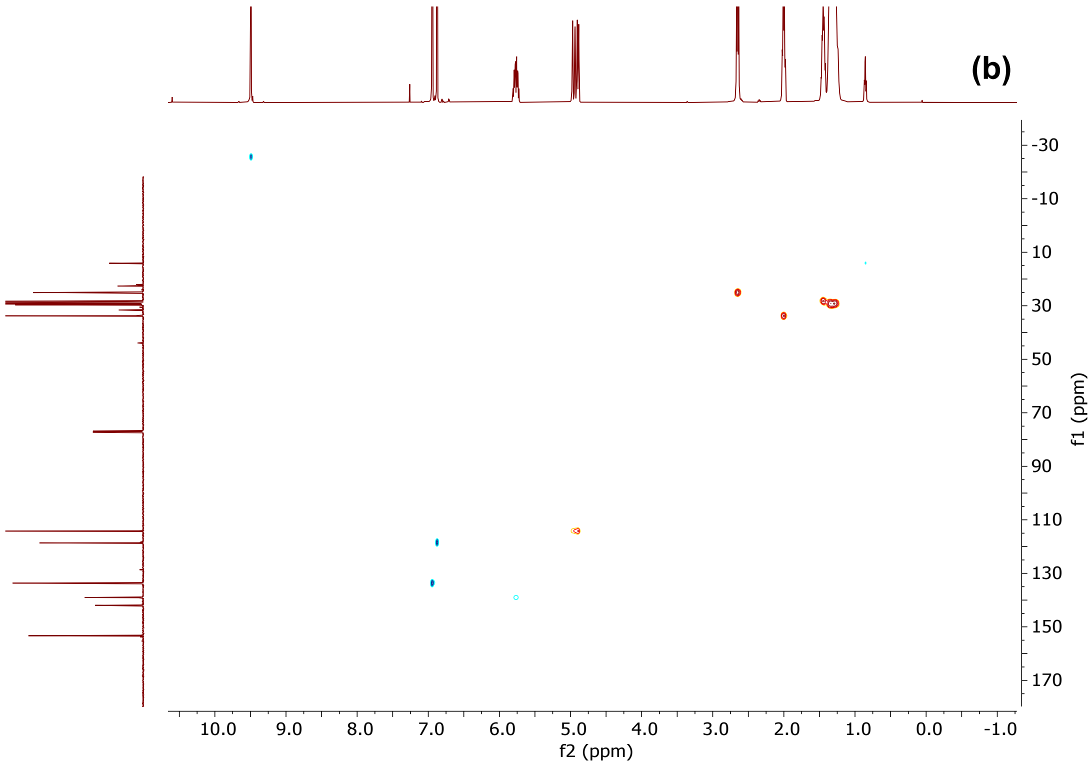

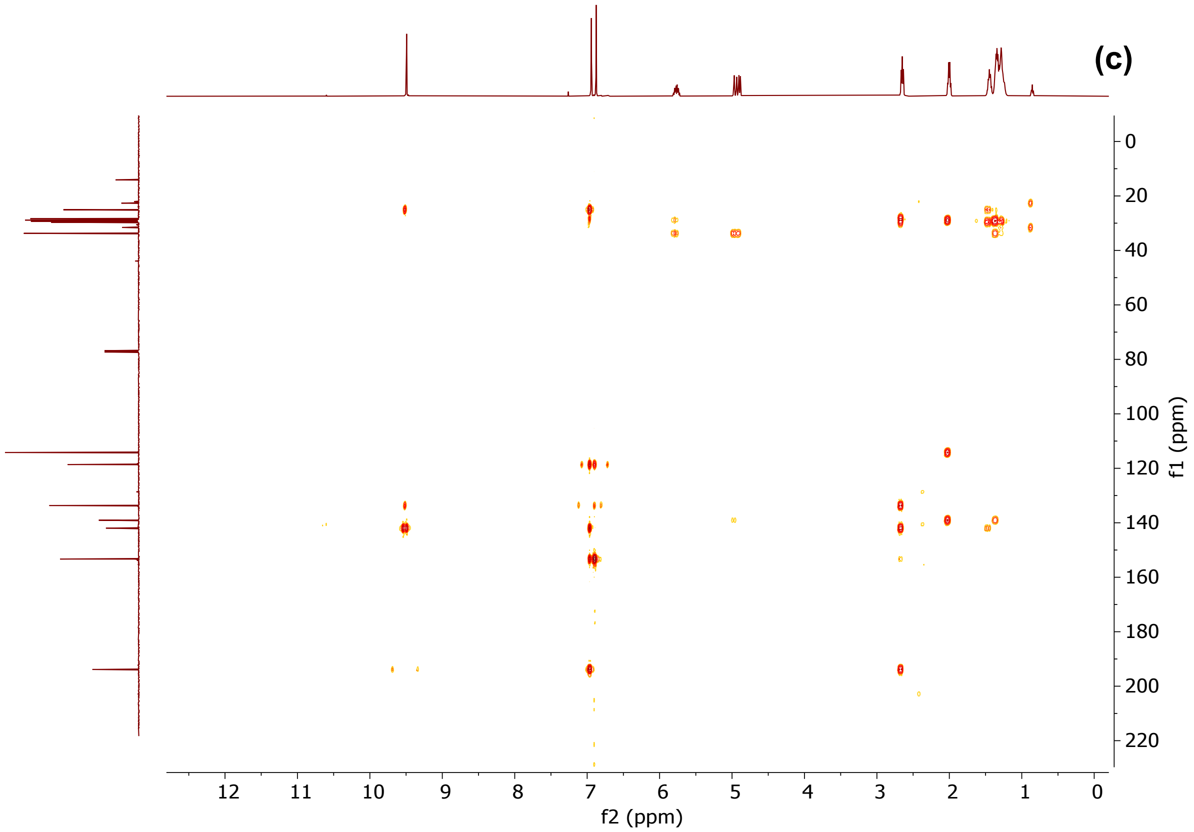

Figure S3. 2D NMR spectra for furan-based α,ω-diene monomer 3; **(a)** COSY **(b)** HSQC **(c)** HMBC.

4.3 Furan-based ADMET polymer

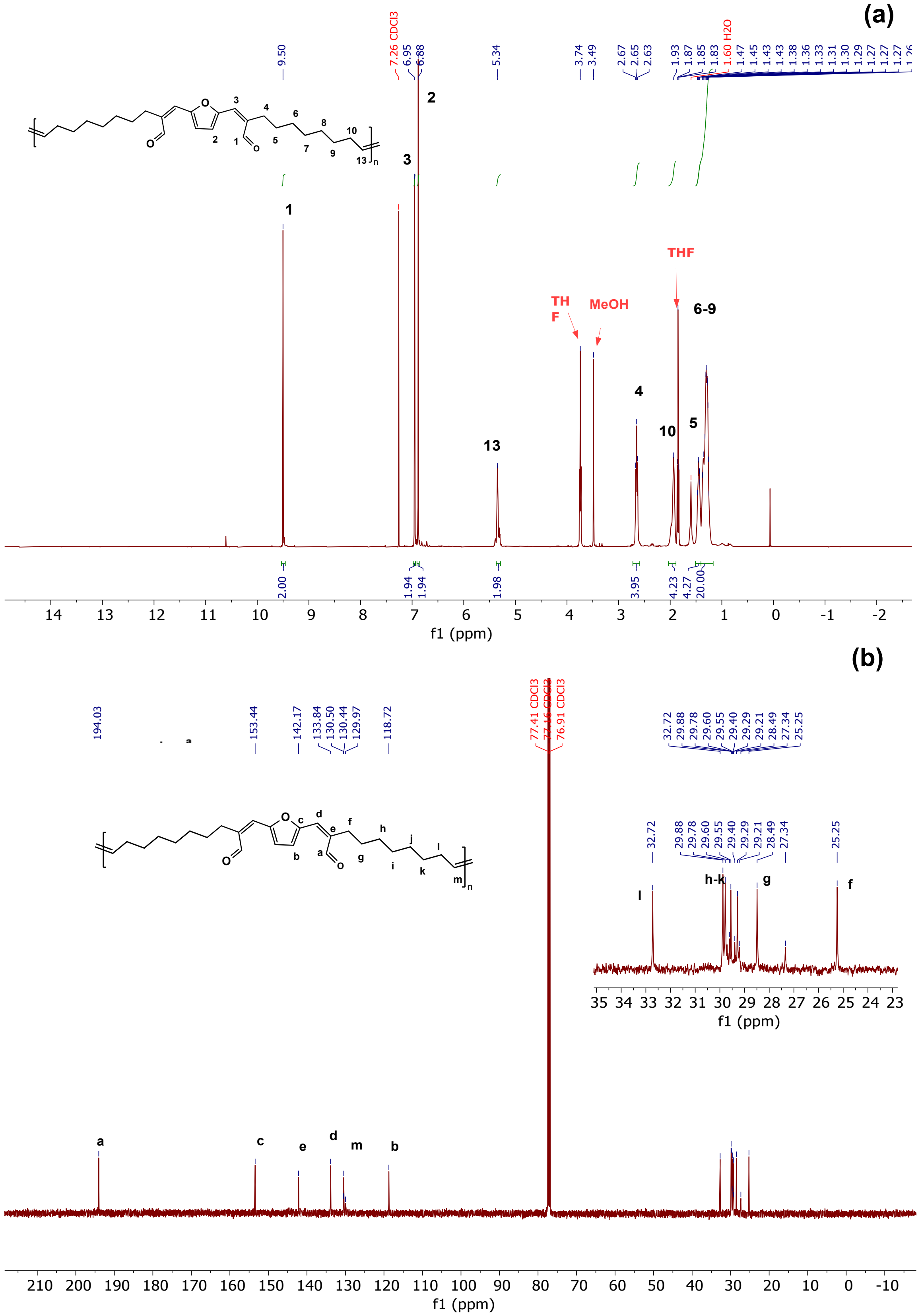

Figure S4. Structural elucidation of polymer produced by ADMET polymerisation of α,ω-diene monomer 3 after purification step; **(a)** ^{1}H NMR **(b)** $\{^{1}H\}$ ^{13}C NMR, performed using $CDCl_3$ at 25°C.

4.4 Double bond isomerization

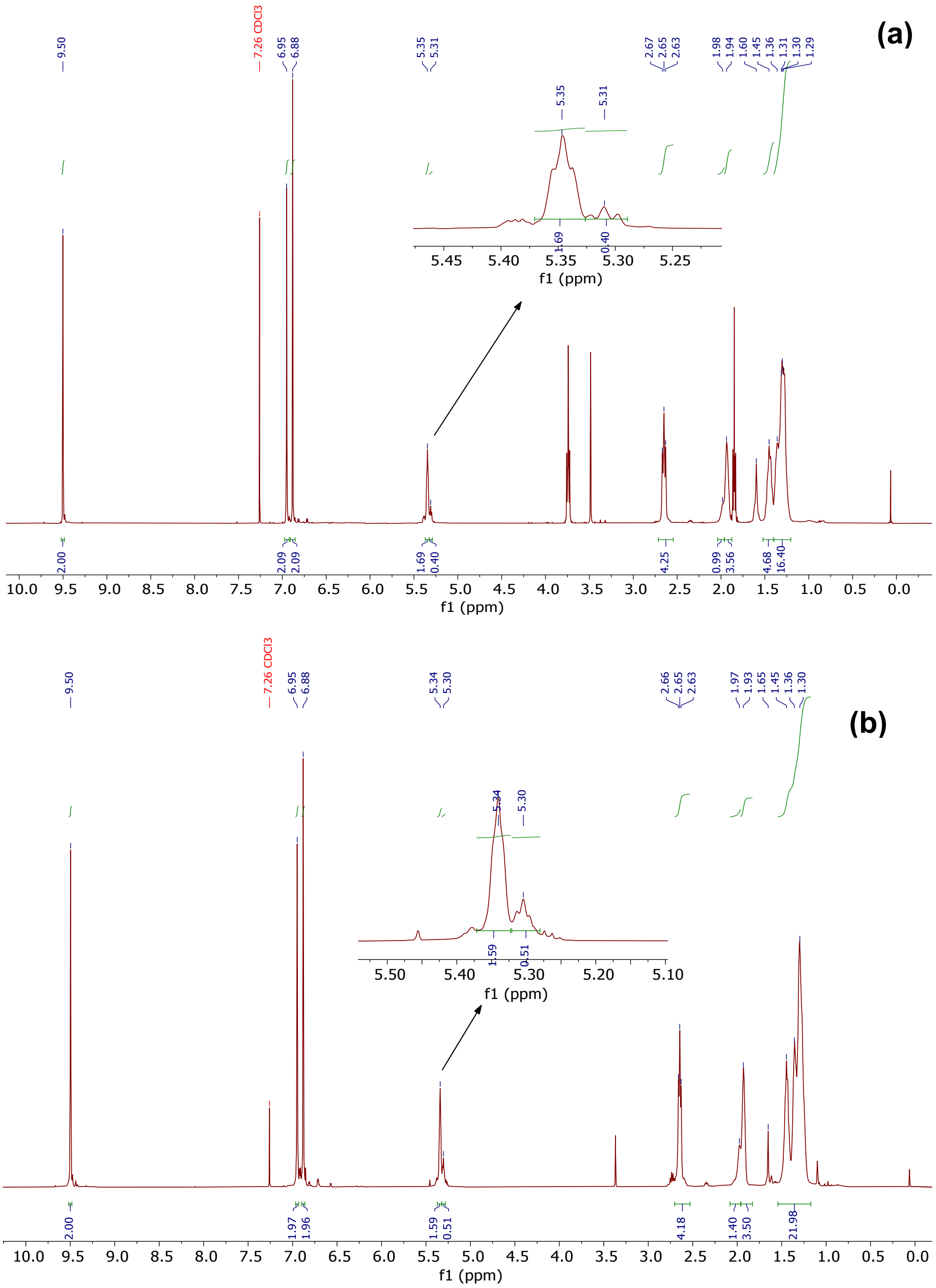

Figure S5. ^{1}H NMR spectra of ADMET polymers produced at different temperatures using GII catalyst (Table 2, main article); (a) PF2 produced at 55 °C (b) PF5 produced at 90°C. The inset spectra show chemical shifts with relative integration for the internal olefin region within the polymer signifying the effect of temperature on the extent of isomerization.

4.5 NMR spectroscopy of TAA-based monomer 5

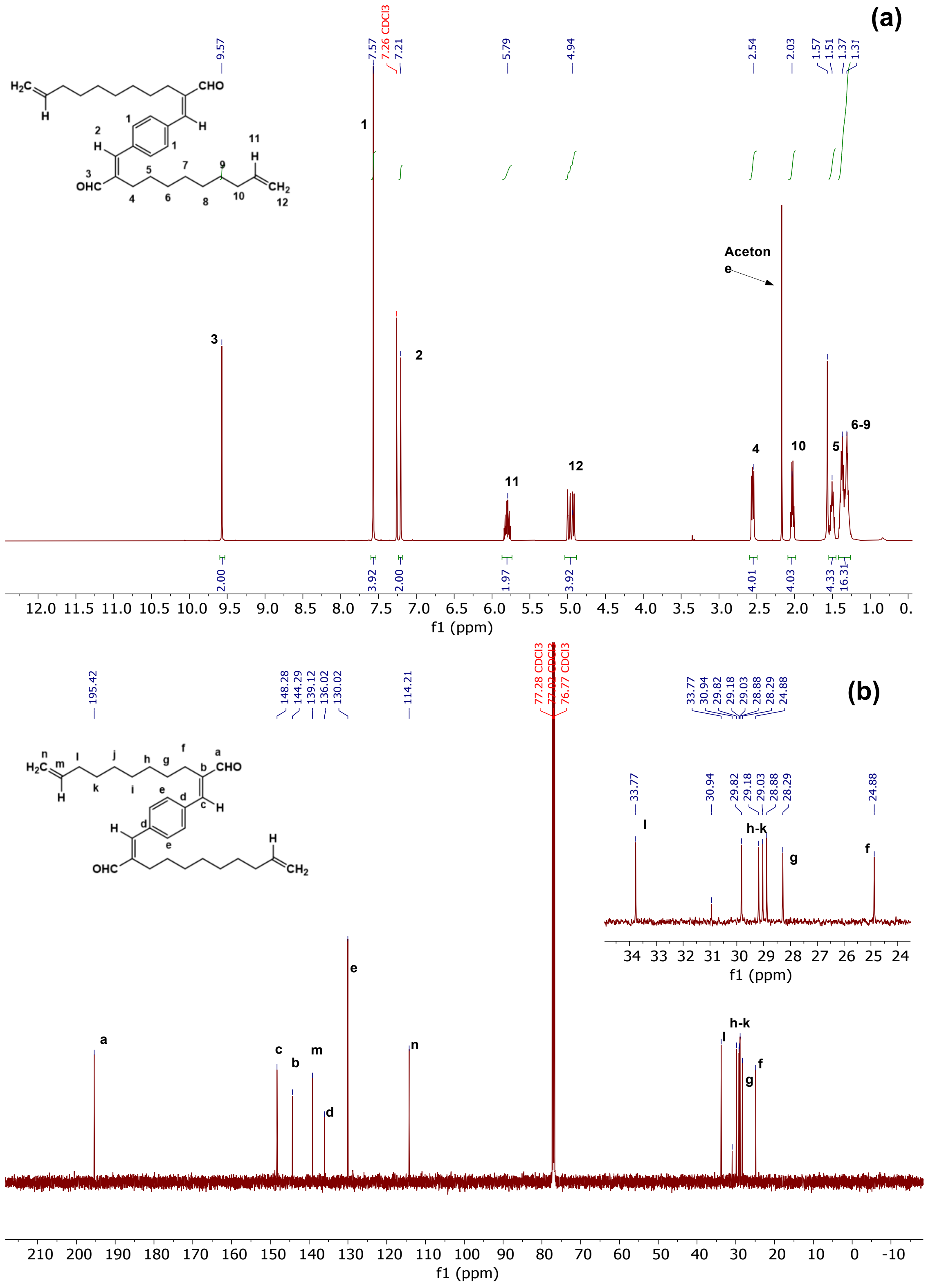

Figure S6. NMR analyses of terephthalaldehyde (TAA) derived α,ω-diene monomer depicting the corresponding assignments conducted in CDCl$_3$ at 25°C; **(a)** ^{1}H NMR **(b)** {^{1}H} ^{13}C NMR.

4.6 ADMET polymer PT1

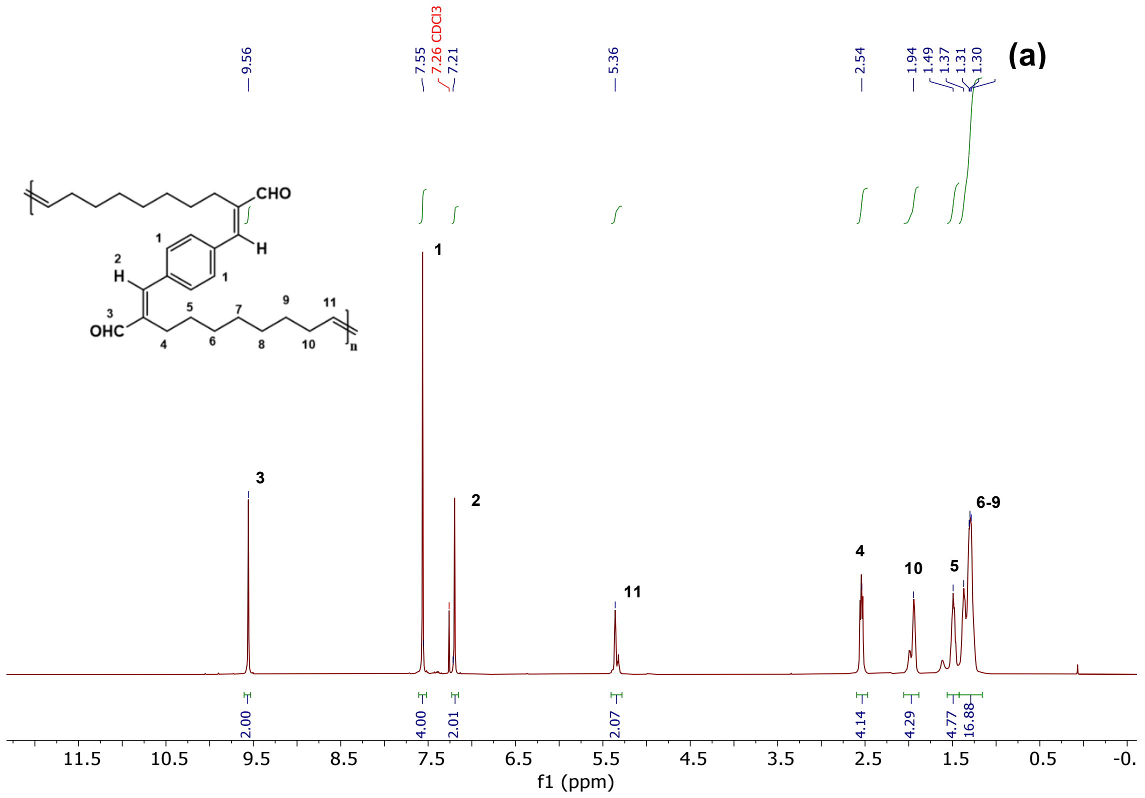

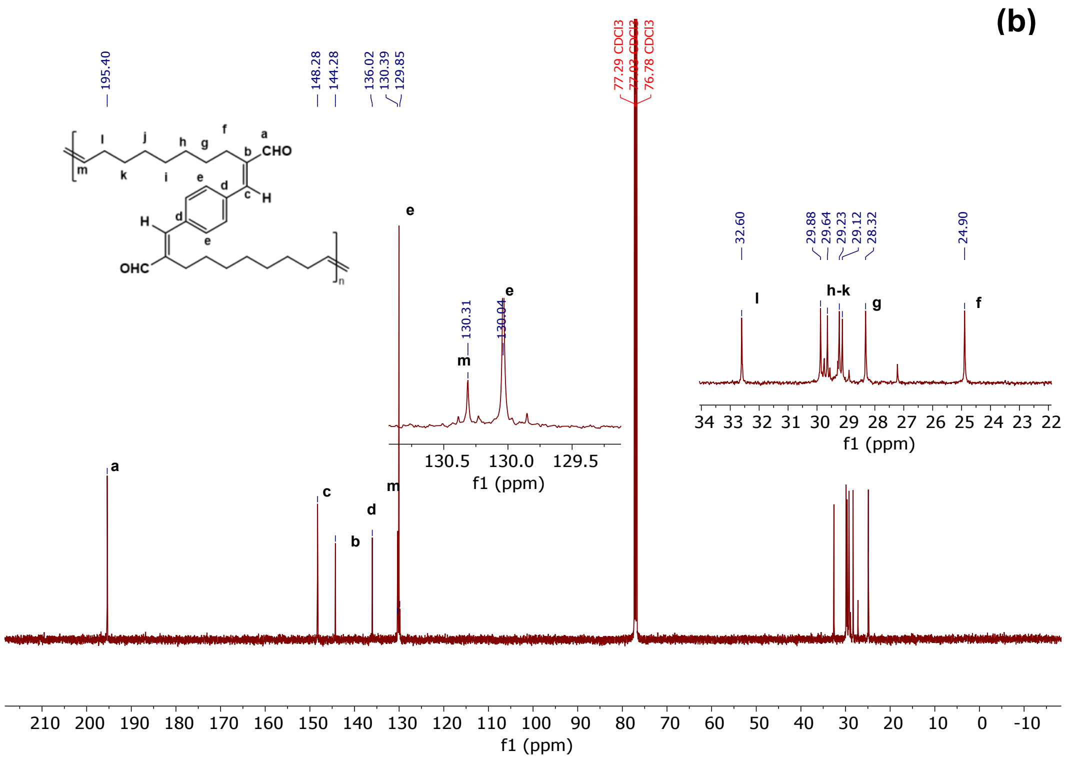

Figure S7. NMR structural assignments for ADMET polymer (PT1) based on terephthalaldehyde (TAA) derived α,ω-diene monomer in $CDCl_3$ at 25 °C; **(a)** ^{1}H NMR **(b)** {^{1}H} ^{13}C NMR.

5 Mass spectrometry

5.1 Mass spectrometry of α,ω-diene monomer 3

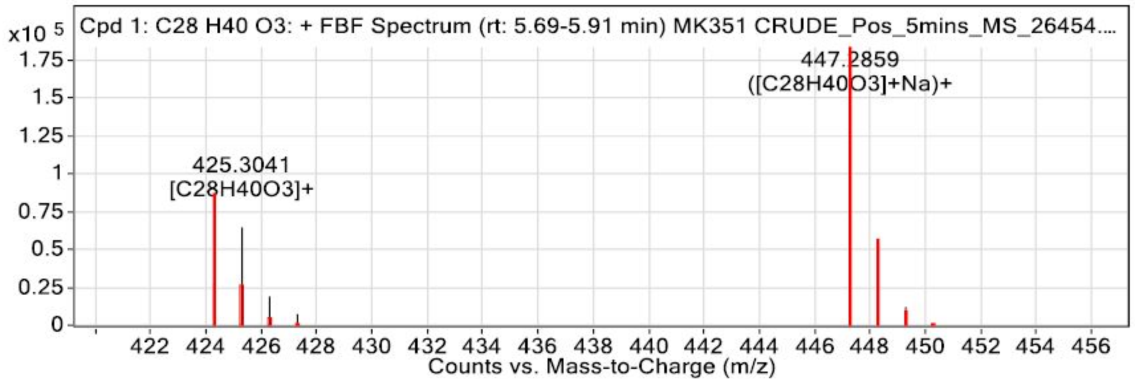

Figure S8. Mass spectrometric analysis of monomer **3** displaying {M+} and {M+Na}$^+$ adducts.

5.2 Mass spectrometry of TAA-based α,ω-diene monomer 5

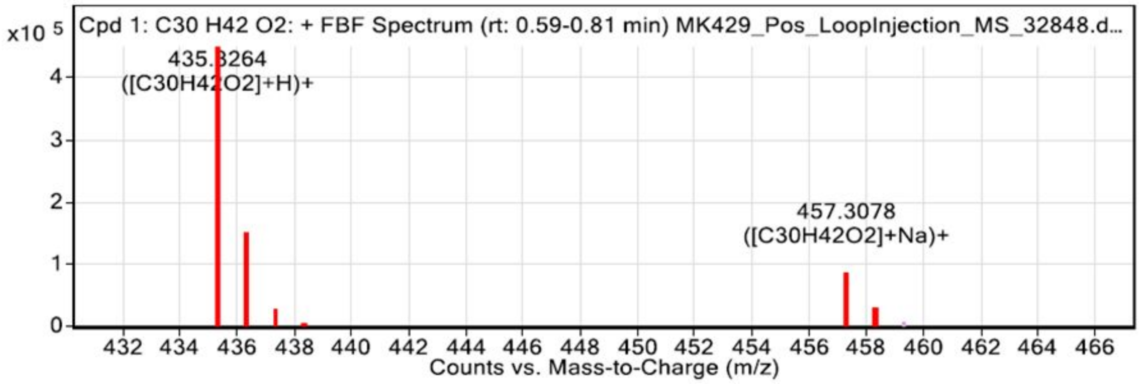

Figure S9. Mass spectrometric analysis results for **5** displaying {M+H}$^+$ and {M+Na}$^+$ adducts

6 GPC traces of ADMET polymers

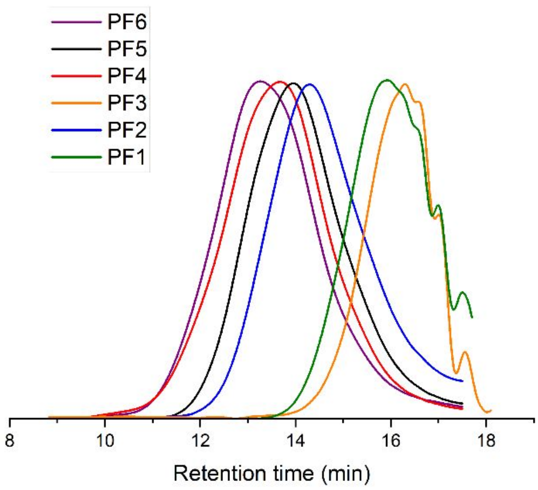

Figure S11. GPC traces of ADMET polymers produced using Grubb's second-generation catalyst (G-II) catalyst.

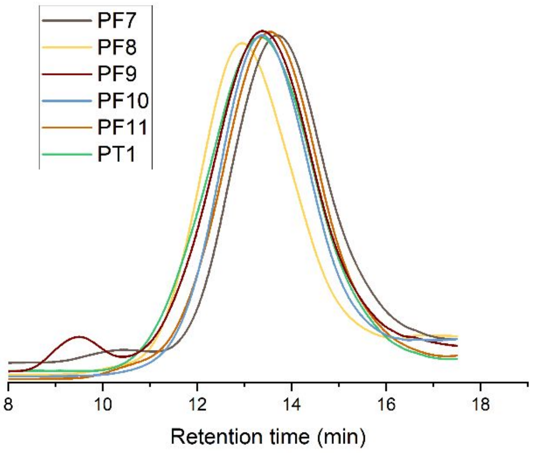

Figure S10. GPC traces of ADMET polymers produced using Hoveyda-Grubbs second-generation catalyst (HG-II) catalyst.

7 DSC analyses curves

7.1 DSC analysis of fully renewable furan-based ADMET polymers

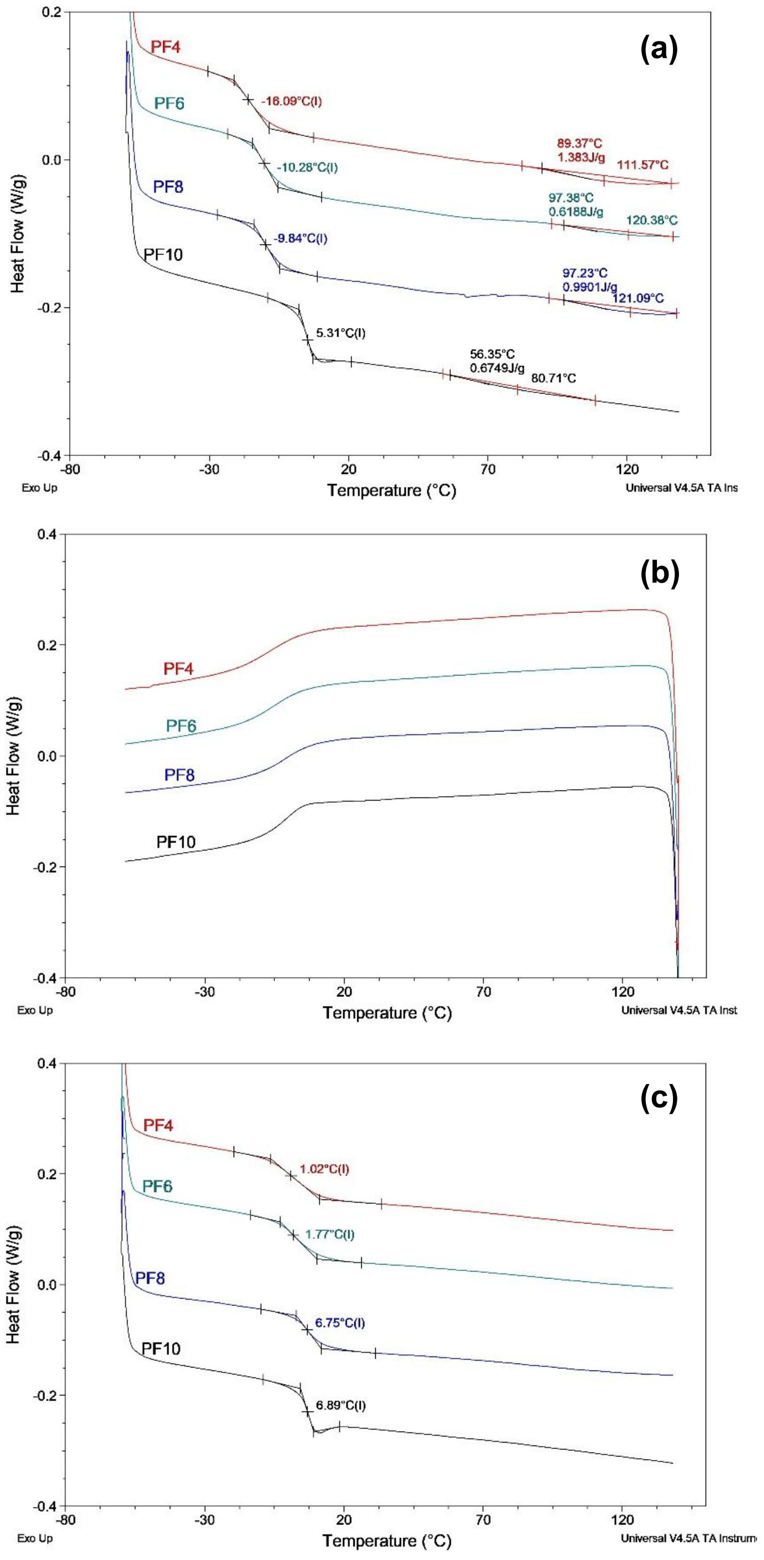

Figure S12. DSC thermograms of selected ADMET polymers; **(a)** first heating scan **(b)** cooling scan **(c)** second heating scan.

7.2 DSC of terephthalaldehyde (TAA) – based ADMET polymer (PT1)

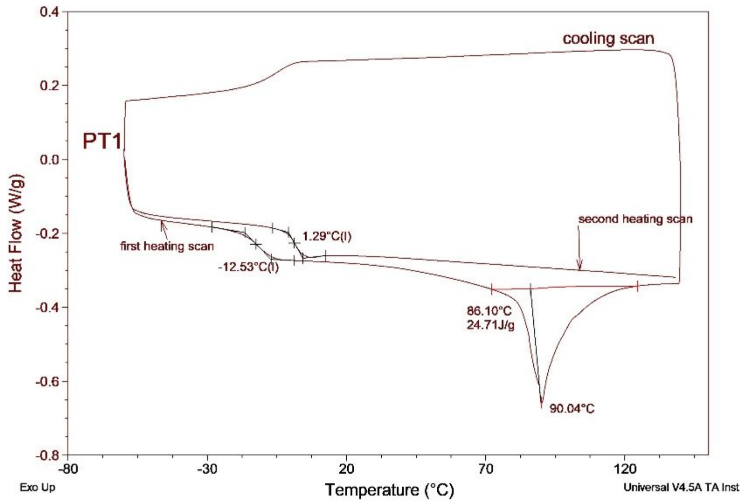

Figure S13. Complete DSC analyses of PT1 polymer.

7.3 DSC thermograms of Furan-based ADMET polymer after modification

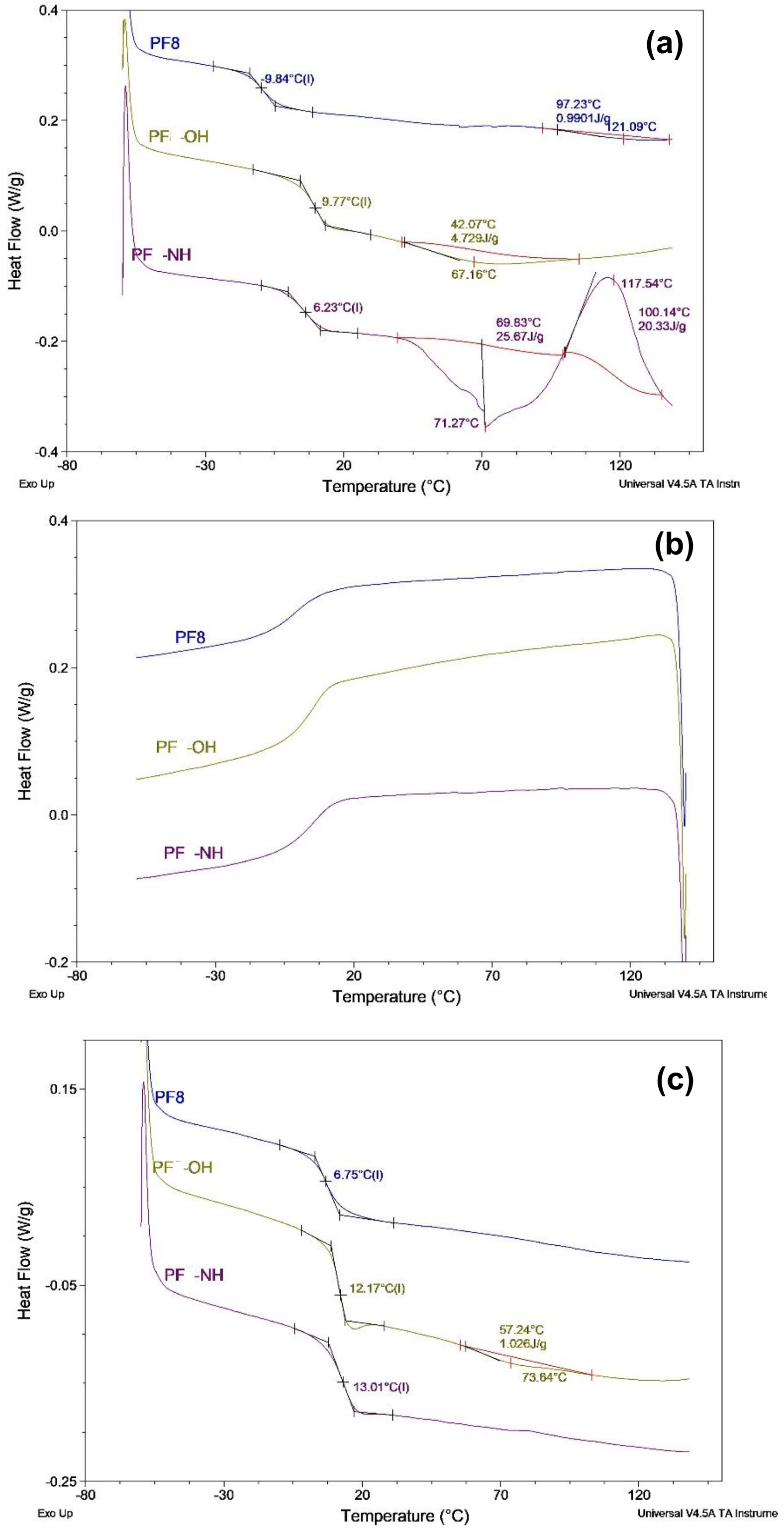

Figure S14. DSC thermograms for furanic polymer PF after post-polymerization modification after reduction (PF-OH) and reductive amination (PF-NH); **(a)** first heating scan **(b)** cooling scan **(c)** second heating scan.

7.4 Polymer hydrophobicity and water contact angle on coated filter paper

Table S3. Water contact angle measured on the coated filter paper surface (average readings for 3 droplets)

Water contact angle for coated paper specimen (°)	S1	S2	S3	Average
	87.4	89.81	91.05	89.42

8 FTIR spectroscopy analyses

8.1 ADMET polymer post-polymerization modification

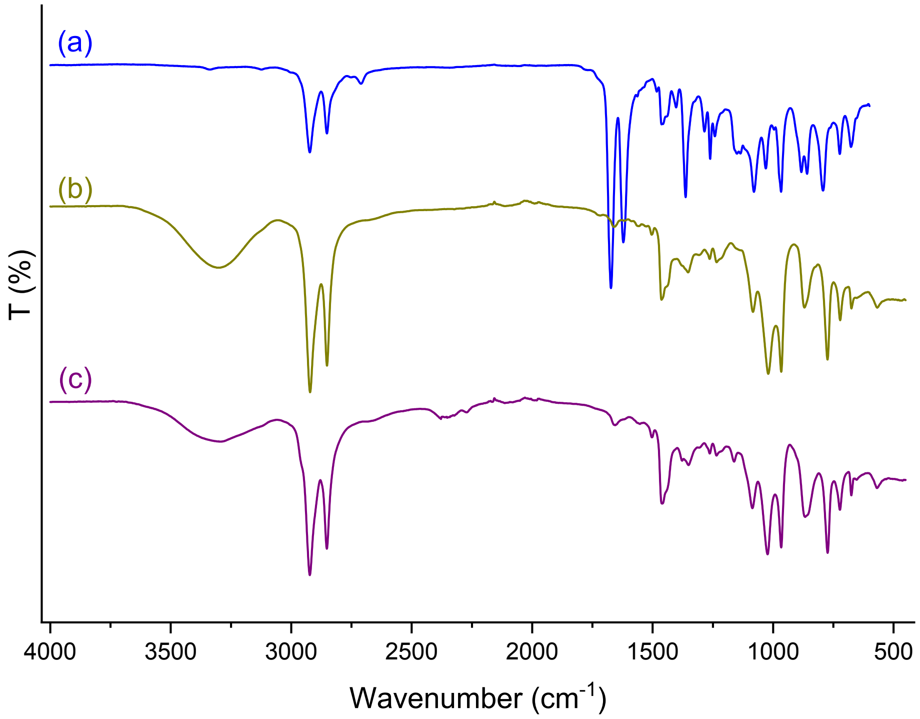

Figure S15. FTIR spectroscopy of ADMET polymer after post-polymerization modification; **(a)** as synthesized polymer **(b)** hydroxyl functional (PF-H) **(c)** amine functional (PF-NH).

8.2 Photoactivity analysis by FTIR spectroscopy

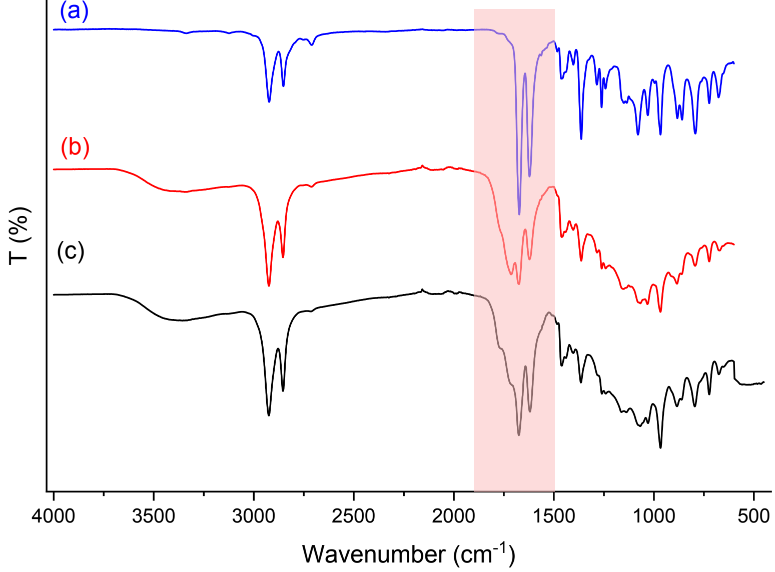

Figure S16. FTIR analysis of ADMET polymers; **(a)** as synthesized **(b)** after UV irradiation for 44 hours **(c)** after storage in visible light at room temperature for 60 days.

9 References

(1) Vijjamarri, S.; Streed, S.; Serum, E. M.; Sibi, M. P.; Du, G. Polymers from Bioderived Resources: Synthesis of Poly(Silylether)s from Furan Derivatives Catalyzed by a Salen-Mn(V) Complex. *ACS Sustain. Chem. Eng.* **2018**, *6* (2), 2491–2497. https://doi.org/10.1021/acssuschemeng.7b03932.

(2) Flourat, A. L.; Annatelli, M.; Fadlallah, S.; Aricò, F.; Allais, F. Biobased Polyethers via Acyclic Diene Metathesis Polymerization of α,ω-Diene Furanics. *Macromolecules* **2023**, *56* (21), 8845–8855. https://doi.org/10.1021/acs.macromol.3c01279.

(3) Wu, Q.; Qin, K. X.; Gan, M. X.; Xu, J.; Li, Z. L.; Li, Z. C. Recyclable Biomass-Derived Polyethylene-Like Materials as Functional Coatings for Commercial Fabrics: Toward Upcycling of Waste Textiles. *ACS Sustain. Chem. Eng.* **2022**, *10* (51), 17187–17197. https://doi.org/10.1021/acssuschemeng.2c05080.

(4) Lillie, L. M.; Tolman, W. B.; Reineke, T. M. Structure/Property Relationships in Copolymers Comprising Renewable Isosorbide, Glucarodilactone, and 2,5-Bis(Hydroxymethyl)Furan Subunits. *Polym. Chem.* **2017**, *8* (24), 3746–3754. https://doi.org/10.1039/c7py00575j.
